# Current Insights into *Sporothrix schenckii*: From Basic Biology to Virulence Mechanisms

**DOI:** 10.3390/jof12010004

**Published:** 2025-12-20

**Authors:** Manuela Gómez-Gaviria, Dario A. Baruch-Martínez, Nathália Faria Reis, Andréa Regina de Souza Baptista, Héctor M. Mora-Montes

**Affiliations:** 1 Departamento de Biología, División de Ciencias Naturales y Exactas, Campus Guanajuato, Universidad de Guanajuato, Noria Alta s/n, col. Noria Alta, C.P., Guanajuato 36050, Guanajuato, Mexico; m.gomezgaviria@ugto.mx (M.G.-G.); da.baruchmartinez@ugto.mx (D.A.B.-M.); 2 Center for Microorganism Research, Biomedical Institute, Fluminense Federal University, Campus Valonguinho-Alameda Barros Terra, S/N, Niterói 24020-150, Rio de Janeiro, Brazil; nathaliafariareis@id.uff.br

**Keywords:** antifungal therapy, epidemiology, fungal cell wall, host–pathogen interaction, sporotrichosis, virulence factors

## Abstract

*Sporothrix schenckii* is a thermodimorphic fungus and one of the main etiological agents of sporotrichosis, a globally distributed subcutaneous mycosis that primarily affects the skin, subcutaneous tissue, and lymphatic system. Historically regarded as the classical species within the *Sporothrix* pathogenic clade, *S. schenckii* remains a clinically relevant pathogen and an important biological model for studying fungal dimorphism, virulence, and host–pathogen interactions. Major virulence factors include melanin production, thermotolerance, hydrolytic enzymes, and adhesins, all of which contribute to its survival and dissemination within the host. Clinically, *S. schenckii* causes a broad spectrum of manifestations ranging from fixed and lymphocutaneous cutaneous forms to disseminated and extracutaneous infections, particularly in immunocompromised individuals. This species exhibits a cosmopolitan distribution with endemic foci in the Americas, Asia, and Africa, and can be transmitted through both sapronotic and zoonotic routes. Diagnosis relies on fungal isolation, molecular identification, and histopathological examination, whereas treatment mainly involves itraconazole, potassium iodide, and amphotericin B for severe cases. This review integrates current knowledge on the biology, virulence, immune response, epidemiology, and treatment of *S. schenckii*, providing an updated overview of its significance as a medically important fungal pathogen with global relevance.

## 1. Introduction

Sporotrichosis is known to be a chronic fungal infection in humans and other mammals, which mainly affects the skin, subcutaneous tissue, and, rarely, internal organs [1,2]. The causative agents of this infection are members of the *Sporothrix* clinical clade, which includes *Sporothrix schenckii*, *Sporothrix brasiliensis*, *Sporothrix luriei*, and *Sporothrix globosa* [3]. These species have different distribution patterns. In the case of *S. brasiliensis*, it is restricted to Brazil and other South American countries, while *S. schenckii* and *S. globosa* have a worldwide distribution. However, reported cases are concentrated in a greater proportion in America and Asia, respectively [4,5,6,7].

Although sporotrichosis is an infection that was described more than a century ago, it is still a neglected infection; the case report is not currently mandatory to report to any National Ministry of Health, except in some Brazilian states [8,9]. Given this, current knowledge of this disease and its various causative agents remains limited compared to other types of mycosis, such as candidiasis, aspergillosis, and cryptococcosis [10].

The increment of cases of human and feline sporotrichosis in recent years, along with the limited information in terms of diagnosis and treatment, has awakened a growing interest from the scientific community to study this infection and its etiological agents [11,12]. Within the pathogenic clade, *S. schenckii* is the most studied species and the one on which most of the available knowledge is concentrated, due to the number of cases documented over time [12]. *S. schenckii* is a thermodimorphic fungus that has historically been considered the main etiological agent of sporotrichosis [13]. Under environmental conditions, it grows as a conidium-producing mycelium, while at 37 °C, within the host tissues, it adopts a yeast-like morphology, a key characteristic in its pathogenicity [14,15]. This species has been isolated from various organic substrates, such as soil, decaying vegetation, and plant thorns, linking it to the classic transmission by traumatic inoculation, although zoonotic forms of transmission, via direct contact with open injuries in infected cats, have also been described, especially in endemic regions [16].

In addition to its thermal adaptability, *S. schenckii* possesses various virulence factors, including hydrolytic enzymes, melanin, adhesins, and cell wall components that contribute to its ability to evade the host’s immune response [17,18]. These characteristics have positioned *S. schenckii* as a valuable model for studying the biology, virulence, and host–pathogen interaction mechanisms of *Sporothrix* species.

Therefore, this review provides an integrated overview of the current knowledge on *S. schenckii*, focusing on its biology, cell wall composition, virulence factors, and the complex interplay with the host immune system.

## 2. Biological Aspects

### 2.1. General Aspects

*Sporothrix schenckii* is one of the main causative agents of human sporotrichosis, a subcutaneous mycosis with the first reported case in 1898 by Benjamin R. Schenck [13,19]. *S. schenckii* belongs to the Ascomycota division, class Pyrenomycetes, order Ophiostomatales, family Ophiostomataceae [13]. It was thought that *S. schenckii* was the sole etiological agent of sporotrichosis; however, taxonomic studies in the early years of this century defined other *Sporothrix* species of clinical relevance: *S. brasiliensis*, *S. globosa*, *Sporothrix mexicana*, and *S. luriei* [14,20]. These species were grouped as the *Sporothrix schenckii* complex [21].

*S. schenckii* is a thermodimorphic fungus, found in soils, plant organic matter, and decomposing organic matter in nature [1]. It has been reported that acquisition of the infection by this pathogen is mainly by traumatic inoculation in outside activities [1] and zoonotic transmission by biting or scratching of infected animals [22].

*S. schenckii* is globally distributed, has been isolated from most of the American countries, including Argentina, Bolivia, Brazil, Colombia, Guatemala, Mexico, Peru, Venezuela, and the United States. In the European continent has been isolated from France, Italy, and the United Kingdom. Finally, in the African and Asian continents, it has been isolated from South Africa, Madagascar, India, China, Thailand, and Japan [4,23,24].

### 2.2. Morphology

**Figure 1:**
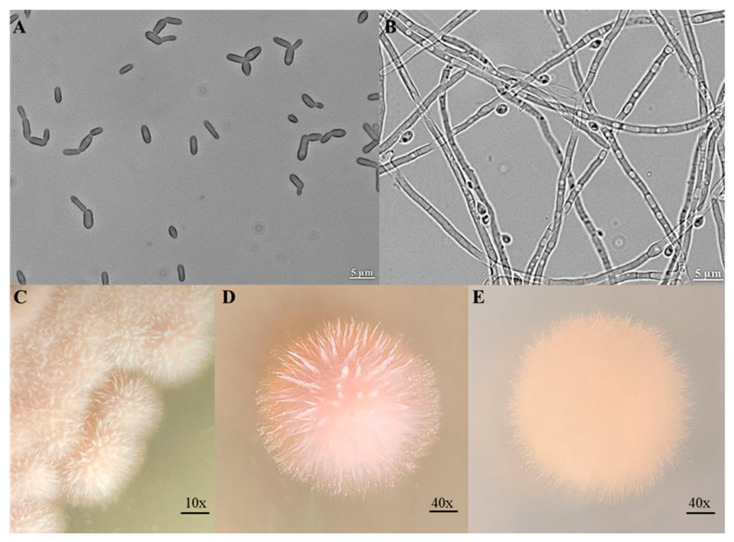
Morphologies of *Sporothrix schenckii* under different culture conditions. (**A**), yeast-like form observed under light microscopy after incubation at 37 °C, showing oval and budding cells. (**B**), mycelial form observed under light microscopy after growth at 25 °C, displaying septate hyphae and conidial structures. (**C**–**E**), macroscopic colony morphology on solid medium at 25 °C. (**C**), 10× view showing cottony texture. (**D**), 40× view of a radial colony with a pinkish center. (**E**), 40× view of a circular colony with homogeneous pigmentation.

*S. schenckii* has a mycelial saprophytic phase with characteristics of slender, hyaline, septate, and branched hyphae that contain conidiophores. These structures form grouped conidia of 2 to 4 µm each, denominated denticles, forming a typical morphology that seems like a flower bouquet [1]. In addition, conidia of this species are known for having a dark cell wall, helping to discriminate *S. schenckii* from other nonpathogenic species [21] (Figure 1A,B).

On the other hand, *S. schenckii* may undergo dimorphism to yeast-like cells, with spindle-shaped or oval cells of 2.5 to 5 µm in diameter, resembling a cigarette [1].

At the macroscopy level, mycelial form can be observed as filamentous colonies, with a white cream color that can turn to brown black after a few days, with a smooth and rough appearance in potato dextrose agar and malt extract. Colonies containing yeast-like cells can be observed as creamy and smooth colonies in Saboraud dextrose agar (Figure 1C–E) [21].

Temperatures of 25 °C and 37 °C for mycelium and yeast-like cells, respectively, are the best to obtain these structures in vitro; however, factors like pH, CO_2_, and the carbon source can affect the growth [25]. In addition, at the molecular level, there are other aspects to consider in the control of dimorphism, such as calcium/calmodulin-dependent protein kinases or a pathway that involves the interaction of a cytosolic phospholipase with a G protein [26,27].

### 2.3. Cell Wall

As in other fungi, the cell wall is a relevant component for *S. schenckii*, as it provides structural integrity and represents the first line of contact with the host. In addition, it works as a molecular scaffold to display various antigenic determinants and virulence factors [28]. In this dimorphic fungus, the cell wall not only plays a protective role but also participates actively in adhesion to host tissues, immune evasion, and modulation of the inflammatory response. Furthermore, its composition varies between the mycelial and yeast phases, reflecting metabolic and structural adaptations to environmental conditions or the host’s internal environment [28].

**Figure 2:**
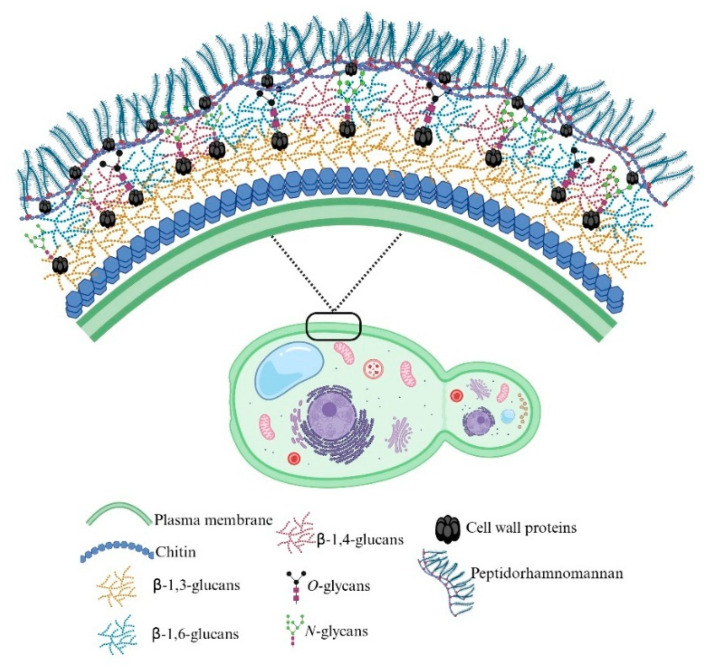
Schematic representation of the *S. schenckii* yeast cell wall composition. This schematic illustrates the multilayered organization of the *S. schenckii* cell wall. The inner layer, located adjacent to the plasma membrane, is mainly composed of structural polysaccharides, such as β-1,3-glucans, β-1,6-glucans, and chitin, which provide mechanical strength and maintain cellular integrity. In contrast, the outer layer contains numerous cell wall proteins, many of which are modified with *N*- and *O*-linked glycans and enriched in mannose residues. A distinctive component of the outermost surface is the peptidorhamnomannan, a complex of glycoproteins rich in rhamnose- and mannose-containing oligosaccharides that is characteristic of *Sporothrix* species.

The latest proposed model for the *S. schenckii* cell wall places in the outermost layer a glycoconjugate called peptidorhamnomannan (PRM), which is composed of several proteins modified with rhamnose- and mannose-containing *O*- and *N*-linked glycans [28,29,30]. The innermost layer contains chitin, β-1,3-glucan, and β-1,6-glucan, which provide rigidity and structural support, while alpha glycogen particles are observed around the plasma membrane, which probably function as energy reservoirs to sustain the synthesis of wall polysaccharides (Figure 2) [31]. Silencing of genes involved in glycosylation pathways, such as *OCH1*, *ROT2*, *MNT1*, and *PMT2*, has revealed notable alterations in wall organization and composition, as well as a reduction in fungal virulence, confirming the relevance of the integrity of this structure for the fungus’s infectious success [32,33,34].

The PRM is composed of more than 300 proteins, some of them likely to be moonlighting peptides [29]. Among the characterized proteins are Pap1 and Hsp60, which have adhesin properties to extracellular matrix (ECM) components [29]. Another relevant cell all protein is Gp70, a glycoprotein with heterogeneous glycosylation patterns, highly antigenic, and also involved in ECM adhesion [31,35,36,37]. These surface proteins not only mediate interaction with the host but can also modulate immune recognition and participate in resistance to cellular defense mechanisms [29,31].

Several studies have shown that environmental and cultivation conditions significantly influence the composition of the cell wall of *S. schenckii*. Cells grown in YP and YNB media showed a significant reduction in the rhamnose and mannose content, with a compensatory increment in the glucan concentration, suggesting that a change in the media can affect the synthesis of oligosaccharides and polysaccharides [38]. In addition, cells showed a 50% reduction in the ability to bind Alcian blue, suggesting defects in the synthesis of both *N*-linked and *O*-linked glycans [38]. These experiments also showed the relevance of cell wall rhamnose-containing glycans for fungal virulence, as cells with low cell wall rhamnose content showed virulence attenuation [38]. This observation was later confirmed by analyzing the cell wall of different *S. schenckii* strains, and those with naturally low rhamnose levels at the cell wall displayed low virulence, when compared with the high cell wall rhamnose-content strains [39].

In addition to nutritional conditions, fungal morphology significantly influences cell wall architecture [40,41]. In the mycelial phase, β-glucans and chitin predominate, while in the yeast phase, the proportion of glycoproteins and PRM increases, which is associated with greater adhesion capacity and immune response evasion [39]. During the temperature-induced morphological transition, *S. schenckii* remodels its cell wall through the coordinated action of glycosyltransferases, chitinases, and glucanases, modifying the exposure of PAMPs and adapting its surface to the parasitic phase. This dynamic process of structural reorganization is a key factor in the thermal virulence and survival of the fungus in the host [39].

### 2.4. Genome

In 2014, *S. schenckii* and *S. brasiliensis* were compared by genomic sequence assemblies and annotations, and the results concluded that both species have a genome identity of 97.5% [42]. Analysis showed that the *S. schenckii* genome is 32.4 megabases (Mb) in size, yielding 16 scaffolds with N50 of 4.3 Mb and 327 contigs [42]. The same study reported 10, 293 protein-coding genes and a G + C content of 62% [42]. These data contrast with the protein-coding genes identified within the *S. brasiliensis* genome, which was significantly smaller (9091 genes) [42]. The mitochondrial genome assembly is about 26.5 Kb with a similarity of 99% with *S. brasiliensis* [42]. Transposable elements (TEs) are present in a proportion of 0.34% in the genome of *S. schenckii*, while in *S. brasiliensis*, a proportion of 0.62%, with two major groups, LINEs and LTRs, and the absence of SINE elements was documented [42]. The proportion of TEs in comparison with other fungi is lower, even with its closely related species, *Paracoccidioides brasiliensis* (TEs of 8–9% of its genome) and similar to others that are not so closely related, such as *Trichoderma* and *Fusarium graminearum* [42]. Even though the intron frequency is similar for both *S. scheckii* and *S. brasiliensis*, in the latter, the intron length is significantly higher (123.4 bd vs. 91.2) [42]. Finally, the analysis of plody in *S. schenckii* indicated that this organism is haploid [43].

## 3. Virulence Factors

In *S. schenckii*, as in other medically important pathogenic fungi, various virulence factors have been described, which contribute to the establishment and progression of infection [18]. These elements are essential during interaction with the host, and their absence can lead to a significant decrease in virulence [44]. Among the most relevant factors reported in *S. schenckii* are adhesins, proteins involved in interaction with ECM components, the ability to form biofilms, the production of hydrolytic enzymes that facilitate tissue invasion, dimorphism and thermotolerance, as adaptation mechanisms, as well as immune evasion strategies, melanin synthesis, and the participation of proteins associated with cell wall structure and remodeling [44]. Knowledge about these factors in *S. schenckii* is still incomplete; however, their identification is essential for understanding the mechanisms underlying fungal pathogenicity. To this end, a BLASTp analysis [45] (https://blast.ncbi.nlm.nih.gov/Blast.cgi, accessed August 2025) was performed using *Candida albicans*, *Aspergillus fumigatus*, and *Cryptococcus neoformans* as reference organisms to predict putative genes in *S. schenckii* associated with virulence factors (Table 1).

Cell adhesion is an initial and decisive step in the fungal infection process, as it facilitates colonization of tissue surfaces and subsequent dissemination of the pathogen within the host [31,46,47]. In pathogenic fungi, surface adhesins, usually proteins anchored to the cell wall, mediate interaction with components of the ECM, such as fibronectin, laminin, collagen, and elastin, promoting the establishment of infection [29,48,49]. In *S. schenckii*, the characterization of specific adhesins is still limited; however, several studies have shown that this fungus has cell wall glycoproteins capable of recognizing and binding to ECM proteins, including fibronectin, laminin, and type II collagen [29,31,49]. These interactions not only promote adhesion to host cells and tissues but also enhance other processes of pathogenesis, such as invasion and evasion of the immune response. The adhesin Gp70 is currently the most studied in the *Sporothrix* complex, and is expressed in *S. schenckii*, *S. brasiliensis*, and *S. globosa* (Table 2) [31,36,50]. In *S. schenckii*, Gp70 is a highly abundant wall glycoprotein. In addition to being attached to the cell wall, it has also been detected as a secreted protein associated with extracellular vesicles, suggesting pleiotropic roles [31,36]. Functionally, Gp70 has been implicated in the adhesion of *S. schenckii* to components of the host ECM. In vitro studies have shown that the purified protein can bind to fibronectin and laminin, and blocking with anti-Gp70 antibodies reduced the fungus’s adhesion to these proteins [31,35]. The generation of *GP70* silencing mutants in *S. schenckii* resulted in a significant decrease in adhesion to laminin and fibrinogen, when compared to the wild-type strain, experimentally confirming its role as an adhesin [31]. Interestingly, the abundance and surface exposure of Gp70 appear to be inversely correlated with the degree of virulence among *S. schenckii* isolates. Highly virulent strains often display reduced Gp70 surface expression, whereas isolates with lower virulence show increased Gp70 density in the cell wall [35]. This inverse relationship suggests that, beyond its adhesive function, Gp70 represents a prominent antigenic determinant. High levels of surface-exposed Gp70 may enhance host immune recognition by promoting antibody binding and opsonization, thereby facilitating the clearance of fungi. In contrast, strains with reduced Gp70 exposure may partially evade immune surveillance, leading to increased virulence. Additionally, strain-dependent differences in Gp70 glycosylation, cell wall organization, and masking by other surface components may further modulate its accessibility and immunogenicity, contributing to the observed variability in virulence [51].

It was recently reported that the chaperonin GroEl/Hsp60 and Pap1 have adhesive properties against various components of ECM, including laminin, elastin, fibrinogen, and fibronectin, while Pap1 also has an affinity for type-I and type-II collagen [29]. Comparative analyses suggest that Pap1 is found in *S. schenckii* and *S. brasiliensis* but is absent in *S. globosa*, which could be related to the lower virulence observed in this species [29]. These findings reinforce the notion that both classic cellular stress proteins, such as GroEl/Hsp60, in moonlighting functions, and recently characterized proteins such as Pap1, expand the repertoire of adhesins in *S. schenckii* and contribute to its ability to interact with the host.

Comparative in silico analyses of the *S. schenckii* genome have identified a set of proteins with a possible adhesin function, mainly glycoproteins anchored to the cell wall by glycosylphosphatidylinositol anchors, predicted using tools such as ProFASTA and FungalRV [42]. Most of these proteins are annotated as hypothetical or belong to families with functions that have not yet been characterized, making it necessary to validate their expression and biological role using proteomic and functional approaches [42]. Several of these candidate adhesins are related to basic fungal cell functions, such as cell wall maintenance, carbohydrate hydrolysis, or hydrolase activity [42]. These findings suggest that, if their role in adhesion is confirmed, many of them would correspond to moonlighting proteins, whose primary function is not directly associated with pathogen-host interaction, but which could contribute to the *S. schenckii* colonization and persistence within the host [54].

Although the experimental characterization of adhesins in *S. schenckii* is still limited, bioinformatics analyses represent a valuable tool for identifying candidates with potential roles in cell adhesion. BLAST(https://blast.ncbi.nlm.nih.gov/Blast.cgi, accessed on 15 August 2025) analysis of the *S. schenckii* genome identified several putative orthologs of adhesins described in reference pathogenic fungi (Table 1). Specifically, no obvious orthologs were found for the *C. albicans* Als family members (Als1–9) or for well-characterized candidate adhesins such as Eap1, Hwp1, or Iff4 (Table 1). However, candidates with high similarity to Ecm33, Int1, and Mp65 proteins, involved in *C. albicans* cell wall interaction and adhesion/biofilm formation were identified [55,56,57]. Analysis against *A. fumigatus* revealed putative orthologs of CalA, Scw11, and Gel1/Gel2, suggesting the presence of multiple enzymes related to cell wall remodeling and polysaccharide processing that could mediate interactions with the host matrix [58,59,60]. Compared to *C. neoformans*, a partial ortholog for Mp98 was detected [61]. In contrast, other adhesins from *A. fumigatus* and *C. neoformans* (RodA, RodB, AspF2, Mp1, AfCalAp, Cfl1, Cpl1) showed no clear orthologs in *S. schenckii* (Table 1). Taken together, these findings point to two relevant ideas: (i) *S. schenckii* possesses a repertoire of proteins related to the cell wall and polysaccharide hydrolysis/modification that could act directly or indirectly as adhesins. However, proteomic and functional validation studies are required to confirm their expression and characterize their actual contribution to the colonization and virulence process in *S. schenckii.* (ii) The absence of obvious orthologs of classical multicentric adhesins suggests that *S. schenckii* has adopted different molecular solutions for adhesion, possibly resorting to proteins with a primary structural/enzymatic function that also perform moonlighting functions in host interaction.

Biofilms are a fundamental component in the biology of numerous fungi, as they promote their persistence in various niches and contribute to pathogenesis [62]. In *Sporothrix* spp., their development begins with cell adhesion to biotic or abiotic surfaces, followed by fungal proliferation accompanied by the production of a polymeric extracellular matrix, and culminates in dispersion to new surfaces [18,62]. These biofilms have a complex architecture characterized by a dense network of hyphae immersed in the extracellular matrix and the formation of aqueous channels, which facilitate both nutrient transport and the structural maintenance of the fungal community [63,64]. Unlike the biofilms of other microorganisms, those of *Sporothrix* spp. exhibit slower growth, which could be related to their three-dimensional organization [63]. Recent evidence suggests that the ability to form biofilms is a key factor in the virulence of the genus, as it confers adaptive advantages against the host and the environment.

In the specific case of *S. schenckii*, biofilm formation has been documented in both in vitro and in ex vivo models, where its filamentous morphology favors the generation of organized structures characterized by intertwined hyphal networks, extracellular matrix, and water channels [64]. In addition, its ability to develop biofilms on keratin-rich surfaces, such as cat nail fragments, has been demonstrated, which is relevant for understanding the mechanisms of sporotrichosis zoonotic transmission [64]. Comparatively, *S. schenckii* exhibits greater metabolic activity in the early stages of biofilm growth than *S. brasiliensis*, although the latter shows greater resistance to antifungal agents in vitro [64]. These findings reinforce the idea that biofilm in *Sporothrix*, and, in particular, in *S. schenckii*, constitutes an essential adaptive strategy that not only favors colonization and transmission but also resistance to adverse environmental and host conditions. Bioinformatic analyses suggest that *S. schenckii* possesses putative orthologs of proteins previously associated with biofilm regulation in other pathogenic fungi (Table 1). This observation, together with experimental evidence of its ability to form biofilms [62,63], indicates that this process could be based on partially conserved molecular mechanisms. However, it is likely that *Sporothrix* has developed additional regulators or adapted them to its biology, underscoring the importance of validating these candidates and identifying new determinants involved in the organization and functionality of its biofilms.

Although factors such as adhesins and biofilm formation have been widely recognized as determinants of pathogenicity in various fungi, dimorphism represents another crucial element in the *Sporothrix* biology [18,65]. This phenomenon allows it to alternate between the saprophytic mycelial phase and the parasitic yeast-like phase, a fundamental transition for its adaptation to the host and dissemination in tissues [65,66]. Transcriptomic studies have shown that this transition involves profound genetic reprogramming, with regulation of pathways related to energy metabolism, cell wall remodeling, oxidative stress response, and cell signaling [42,66]. Among the molecules involved are heat shock proteins (Hsp70, Hsp90) and the histidine kinase SsDRK1, homologous to factors described in other dimorphic fungi, underscoring the conservation of molecular mechanisms associated with dimorphism [65,66,67]. In addition, calcium has been shown to play a central role in morphological transition by stimulating calmodulin-dependent signaling pathways (CaMK). In this context, the protein kinase Sscmk1 has been identified as an essential regulator of morphogenetic and proliferative processes in *S. schenckii* (Table 2) [53]. This evidence confirms that dimorphism in *S. schenckii* is a multifactorial and finely regulated process, the elucidation of which remains fundamental to understanding the biology and pathogenesis of the *Sporothrix* genus.

Although some molecular regulators of dimorphism in *S. schenckii* are known, information is still limited, and further experimental studies are needed to fully elucidate the proteins involved in this process. In *C. albicans*, regulators such as Cph1, Hgc1, Nrg1, and Tup1 play central roles in the morphological transition, controlling hyphal formation and the repression of genes associated with the yeast phase [68,69,70]. According to our bioinformatic analysis, the *S. schenckii* genome contains putative orthologs for these genes, suggesting the conservation of key regulatory pathways (Table 1). Similarly, in *C. neoformans*, proteins such as Mob2, Cbk1, Tao3, and Sog2 participate in the RAM (Regulation of Ace2 and Morphogenesis) pathway, which is involved in cell organization and polarity [71,72]. Our analysis identified orthologous candidates in *S. schenckii*, indicating that this fungus may share conserved regulatory elements with other dimorphic pathogens. These results suggest that the control of dimorphism in *S. schenckii* likely involves a network of transcriptional and signaling factors that are partially conserved with *C. albicans* and *C. neoformans* but also include genes that have not yet been characterized and that could be adapted to the biology of this genus.

Given that morphological transition is closely related to environmental conditions, the ability to grow and survive at 37 °C becomes an indispensable prerequisite for completing this process [47]. Thus, thermotolerance is not only necessary for the establishment of the parasitic yeast phase but also represents an essential virulence factor in *Sporothrix* [47]. Thermotolerance is an indispensable trait for the adaptation of pathogenic fungi to the host. As in other dimorphic fungi, the response to increased temperature activates a series of cellular protection mechanisms that include heat shock proteins, calcium-dependent signaling pathways, and cell wall remodeling, all of which are essential for maintaining viability and infectivity [66]. This property has been considered a key virulence factor, as strains unable to sustain growth at physiological temperatures show marked attenuation in experimental models, highlighting the close relationship between thermotolerance, dimorphism, and pathogenesis in *Sporothrix* [47]. Likewise, clinical isolates from cutaneous and disseminated forms have shown efficient growth between 37 °C and 38 °C, indicating that most strains of *S. schenckii* possess effective thermotolerance [47]. At the molecular level, chaperone proteins, such as Hsp90 and kinases, such as Sscmk1, actively participate in the response to heat stress, regulating homeostasis and morphological maintenance at physiological temperatures [26]. However, this capacity has some limitations, with optimal growth of *S. schenckii* observed between 20 °C and 30 °C, decreasing significantly above 40 °C [1]. Taken together, these findings reinforce that thermotolerance is a dynamic and highly regulated mechanism, essential for the adaptation of *S. schenckii* to the host environment and closely linked to its morphogenetic and virulence capacity.

Knowledge about the molecular mechanisms that regulate heat tolerance in *S. schenckii* is still limited. However, BLASTp analysis allowed us to identify several putative orthologs of genes involved in the heat response of reference pathogenic fungi (Table 1). Orthologs of the heat shock proteins Hsp60, Hsp104, Ssa1, and Ssb1 from *C. albicans* were detected, all of which are involved in maintaining protein homeostasis and cell survival under heat stress [73,74]. Similarly, orthologs of *A. fumigatus*, corresponding to CrgA, Sch9, Hsf1, BiP/Kar2, Ssc70, Hsp88, Lhs1/Orp150, and Hsp90 were identified, which perform essential functions in the regulation of protein folding, cell signaling, and response to environmental stress conditions [75,76]. In addition, an ortholog of Ccr4 described in *C. neoformans* was found, related to transcriptional regulation and adaptation to adverse thermal conditions [77]. Among these genes, Hsp90 stands out, which has been experimentally characterized in *S. schenckii* and whose participation is crucial for thermal tolerance and maintenance of the yeast phase at 37 °C [26]. The presence of orthologs of these and other conserved genes reinforces the hypothesis that *S. schenckii* uses a molecular repertoire like that of other dimorphic fungi, supported mainly by heat shock proteins and signaling regulators, to resist the physiological conditions of the host.

Hydrolase production (proteases, lipases, phospholipases, among others) is a mechanism frequently associated with the invasion and spread of pathogenic fungi to the host [78]. In *S. schenckii*, although this field has been less researched, there is evidence that these enzymes contribute to cell damage and tissue adaptation [79]. For example, studies have shown that fungal proteases can induce cytopathic effects in human epithelial cells, using assays with substrates, such as azocoll and zymograms, and that their activity is maintained at different pH levels (5 and 7) [79]. In addition, *S. schenckii* strains have shown lipolytic activity with different substrates (olive oil, Rhodamine B, or Tween 80), suggesting that secreted lipases could participate in the degradation of host lipids and adaptation to infection niches [80]. Proteomic assays of the cell wall under oxidative stress conditions have also revealed secreted or extracellular proteins that could have hydrolytic or wall remodeling functions as part of the response to the host environment. These observations support the hypothesis that *S. schenckii* deploys a battery of hydrolytic enzymes as part of its virulence strategy [52,79,81].

Based on the bioinformatic analysis performed, it was possible to identify several putative orthologs of proteins reported in *C. albicans* and *A. fumigatus* associated with the production of hydrolytic enzymes, such as phospholipases, lipases, and proteases, in the *S. schenckii* genome (Table 1). Orthologs of the Lip5-8, Sap1-8, and Plb1-3 proteins from *C. albicans* were found, as well as Pep1, Pep2, Ap1, CtsD, and PlaA from *A. fumigatus* [82,83]. These proteins perform key functions in other pathogenic fungi, such as the degradation of host components, tissue invasion, and evasion of the immune response [84].

Other virulence factors play an important role in the biology of pathogenic fungi, including immune evasion, melanin production, and cell wall synthesis. About immune evasion, *S. schenckii* has developed various strategies that allow it to survive and establish itself within the host, even in the face of a phagocytic cell response. This process involves coordinated mechanisms, such as biofilm formation, protease secretion, morphological changes, and the synthesis of proteins with immunomodulatory functions [47,85]. The mechanisms described include the secretion of molecules that interfere with complement activation, structural modification of cell wall components, such as differential exposure of β-glucans and glycoproteins, and the release of extracellular vesicles that transport antigens and immune recognition-modulating enzymes [47,85]. These strategies reduce detection by pattern recognition receptors, such as dectin-1 and TLR2, favoring fungal persistence in infected tissues. Proteomic studies have identified several proteins related to these processes in the *S. schenckii* yeast-like phase, including aminopeptidase I, manganese superoxide dismutase, 70 kDa heat shock protein (Hsp70), glyceraldehyde-3-phosphate dehydrogenase (GAPDH), hydroxymethylglutaryl-CoA lyase, acetyl-CoA hydrolase, and 3-oxoacyl-[acyl carrier protein] reductase [42,86]. These proteins play key roles in the dimorphic transition, extracellular vesicle production, protection against oxidative stress, and tissue invasion. Aminopeptidase I could contribute to the weakening of host defenses, while 3-oxoacyl reductase, involved in rhamnolipid biosynthesis, has been associated with direct interaction with immune cells [86]. Likewise, surface proteins, such as Gp70 and Hsp60, in addition to their adhesive role, act as immunodominant antigens capable of modulating the activation of immune cells, facilitating the evasion of the immune response and the persistence of the fungus in the host [29,31,35]. Although several proteins potentially involved in the immune evasion of *S. schenckii* have been identified, further experimental evidence is still needed to fully understand the complex network of interactions that allows it to resist the action of the host’s immune system. The BLASTp analysis performed in this study contributes to expanding knowledge about this process by revealing the presence of putative orthologs of genes known for their involvement in immune evasion in other pathogenic fungi. These include Hgt1, Msb2, and Sit1 from *C. albicans*, PksP/Alb1 from *A. fumigatus*, and Rim101 from *C. neoformans* (Table 1).

In addition to mechanisms that enable evasion of the immune response, other factors contribute significantly to *S. schenckii*’s ability to persist and cause infection in the host. These include melanin production and cell wall synthesis and remodeling, two closely related processes that provide physical and chemical protection against immune system defenses and adverse environmental conditions [87,88]. Melanin is a pigment associated with the cell wall that acts as an important virulence factor in *S. schenckii*, conferring protection against the host’s immune response and adverse environmental conditions [47,87]. This polymer can mask immune recognition epitopes and neutralize reactive oxygen and nitrogen species, contributing to the survival of the fungus during infection [89]. In *S. schenckii*, the production of at least three types of melanin has been described: DHN-melanin, eumelanin, and pyomelanin, each synthesized by distinct but functionally complementary biosynthetic pathways [89,90]. In addition to its role in immune evasion, melanin also confers resistance to antifungal agents such as amphotericin B and terbinafine, as well as to nitrogen-derived oxidants [89].

Comparative studies have shown that *S. schenckii* produces detectable amounts of DHN-melanin in both its mycelial and yeast-like phases, although in lower proportions than *S. brasiliensis*, which could be related to the differences in virulence observed between these species [47,89,91]. The bioinformatics analysis performed in this study identified putative protein orthologs in *S. schenckii* associated with melanin synthesis, previously characterized in *A. fumigatus*, such as Fet3 oxidase and phenoloxidase-type enzymes, all involved in the polymerization and deposition of the pigment within the cell wall (Table 1).

Complementarily, cell wall synthesis and remodeling in *S. schenckii* constitute another essential component of its virulence. The wall, composed mainly of chitin, β-1,3-glucan, β-1,6-glucan, and PRM, undergoes a profound structural reorganization during the mycelium-yeast transition, which modifies the exposure of immunogenic determinants and affects recognition by host receptors, such as dectin-1 and TLR2 [29,39,40,92]. In this regard, proteins involved in wall biosynthesis and assembly, such as chitin synthases, glucanases, and GPI-anchored proteins, could function dually, participating in both cell architecture and host interaction [93].

Taken together, the virulence factors described reflect that *S. schenckii* has a remarkable ability to adapt to the host, supported by coordinated mechanisms that include adhesion, dimorphism, thermotolerance, melanization, and cell wall remodeling. Although its virulence is considered intermediate within the pathogenic clade, compared to *S. brasiliensis*, the differences seem to lie less in the presence or absence of specific factors and more in their level of regulation and functional efficiency [47,66]. In this sense, *S. brasiliensis* exhibits greater expression of genes associated with energy metabolism, oxidative stress, and melanin production, which could confer advantages in colonization and tissue dissemination [42]. However, *S. schenckii* maintains a sufficient repertoire of strategies to cause both cutaneous and disseminated infections, demonstrating physiological plasticity that ensures its persistence in various environments and hosts [47]. Understanding the molecular and functional variations between the two species will be key to clarifying the evolutionary determinants of virulence within the *Sporothrix* genus.

## 4. Immune Response Against *S. schenckii*

During the interaction between *S. schenckii* and the host, the innate immune response plays a fundamental role in controlling infection [94]. This process begins with the recognition of PAMPs by pattern recognition receptors (PRRs) expressed in immune system cells [28,95]. PAMPs are conserved structures that the host cannot synthesize and are present in various microorganisms, including pathogenic fungi. In *S. schenckii*, the cell wall is the main source of these PAMPs, as it is composed of chitin, β-glucans, melanin, glycoproteins, and PRM. These components act as recognizable signals for the immune system and trigger the activation of initial inflammatory responses [39,40,41,96]. Among them, PRM, rich in mannose and rhamnose residues, has been identified as a key PAMP in immune recognition due to its ability to activate C-type lectin receptors (CLRs) and Toll-like receptors (TLRs) and promote the release of proinflammatory cytokines [97,98]. In addition, it has been shown that the structure and morphology of the fungus directly influence its recognition by the host [41,92,99]. Variations in the exposure of wall polysaccharides can modify the activation of PRRs, affecting the efficacy of the innate response and conditioning the subsequent induction of adaptive immunity [100].

The recognition of *S. schenckii* cell wall components is mediated by a wide variety of PRRs expressed mainly in macrophages, dendritic cells, neutrophils, and human peripheral blood mononuclear cells (PBMCs) [41,92,96,97,99]. Among these, TLRs and CLRs play a central role [101,102,103]. Several studies have shown that TLR2 and TLR4 recognize components of the fungal wall and contribute to the production of proinflammatory cytokines, such as TNF-α, IL-6, and IL-1β, which promote the recruitment of immune cells to the infection site [101,103,104]. For their part, CLRs, especially dectin-1, recognize β-1,3-glucan residues, activating Syk- and NF-κB-dependent signaling pathways, which promote the release of inflammatory mediators and the activation of adaptive immunity [41,97,105]. Together, the coordinated activation of these receptors allows for the early detection of *S. schenckii* and the initiation of effector mechanisms that limit its spread [97]. In parallel, activation of the complement system, particularly the C3 component, facilitates opsonization of the fungus and enhances phagocytosis by macrophages and neutrophils, thus reinforcing early defense mechanisms [94,100].

Once PRRs detect *S. schenckii* components, multiple innate immunity effector pathways are activated, involving the participation of phagocytic cells such as macrophages and granulocytes [92,99,102,106,107]. In particular, neutrophils play a crucial role in the early stages of infection. It has recently been shown that different fungal morphologies, conidia, germlings, and yeast-like cells induce different responses in human granulocytes, modulating phagocytosis, cytokine release, and the formation of neutrophil extracellular traps (NETs) [92]. The *S. schenckii* yeast-like cells stimulate vigorous NET formation, and β-1,3-glucan, as well as *N*- and *O*-glycans present in the cell wall, were identified as the main PAMPs for interaction with granulocytes [92]. In addition, it was observed that different species of the *Sporothrix* genus use different recognition pathways. In *S. globosa*, the dectin-1-dependent pathway predominates, while in *S. brasiliensis*, detection occurs mainly through TLR4 and the complement receptor CR3. This suggests that the composition of the cell wall determines the recognition mechanism and the magnitude of the neutrophilic response [92].

Complementarily, PBMCs also actively participate in the initial stages of *S. schenckii* infection. These cells recognize fungal wall components, including β-1,3-glucans, *N*-glycans, and *O*-glycans through receptors such as dectin-1, TLR2, and TLR4, triggering the production of proinflammatory cytokines, such as TNF-α, IL-6, and IL-1β [41]. The elimination of *O*-glycans in *S. schenckii* conidia significantly reduced the production of these cytokines, confirming the immunomodulatory role of these sugars in the fungus-host interaction. Furthermore, the magnitude of activation varies according to the fungus morphology, with yeast-like cells stimulating the most robust cytokine response [41,99].

Macrophages constitute one of the main lines of defense against *S. schenckii*, as they perform phagocytosis functions and produce microbicidal mediators [96,102,107]. After recognizing the fungus through TLR2, TLR4, and dectin-1, these cells release proinflammatory cytokines, such as TNF-α, IL-1β, IL-6, and IL-12, which promote the recruitment and activation of other immune cells [96,108]. Phagocytosis is enhanced by opsonization with antibodies or complement components, which increases the activation of Fc and CR3 receptors and amplifies the production of inflammatory mediators [96,97]. During this process, macrophages generate reactive oxygen species (ROS) and nitric oxide (NO), which are essential for controlling infection [109]. However, *S. schenckii* can partially resist these mechanisms by producing melanin and reorganizing its cell wall, strategies that reduce oxidative stress and allow its intracellular persistence [110]. Murine models with deficient ROS production show a significant decrease in the ability of macrophages and neutrophils to eliminate *S. schenckii*, highlighting the importance of oxidative mechanisms in the early fungus containment [109,111,112].

It has also been identified that Gp70 on the surface of *S. schenckii* acts as a target for antibodies that facilitate opsonization and macrophage-mediated phagocytosis, evidencing the cooperation between the cellular and humoral components of the immune response [31,106]. Human monocyte-derived dendritic cells also actively participate in the recognition of *S. schenckii* and in the link between innate and adaptive immunity. It has been shown that phagocytosis of conidia and yeast-like cells depends on receptors such as the mannose receptor, complement receptor CR3, DC-SIGN, and TLR4, which cooperate in the internalization of the fungus [96]. The elimination of *N*- and *O*-glycans from the wall significantly reduced fungal uptake, suggesting that the organization of glycans on the surface is critical for their recognition. Once activated, these cells secrete proinflammatory cytokines, including TNF-α, IL-6, IL-1β, and IL-12 in proportions that vary according to fungal morphology, promoting an environment that favors the differentiation of Th1 and Th17 lymphocytes in the early stages of infection [96,113].

After initial activation, the adaptive immune response plays a decisive role in controlling and resolving the *S. schenckii* infection. The activation of CD4^+^ T lymphocytes occurs after the presentation of fungal antigens, in the context of the major histocompatibility complex class II (MHC-II), which induces their differentiation into effector subpopulations with specialized functions [114]. In both experimental models and in patients, it has been shown that a Th1-type response characterized by the production of IFN-γ and TNF-α is essential for macrophage activation and intracellular elimination of the fungus, while a Th17 profile, mediated by IL-17 and IL-22, contributes to neutrophil recruitment and early control of infection [100,115,116]. In contrast, a predominantly Th2 response, associated with the production of IL-4 and IL-10, is linked to reduced fungicidal capacity and a more severe disease progression. Taken together, these findings confirm that the balance between Th1 and Th17 responses determines the outcome of infection and underscore the relevance of interactions between innate and adaptive immunity [100].

In addition to T cell-mediated cellular immunity, the humoral response also plays an important role in defense against *S. schenckii* [117,118]. Specific antibodies targeting cell wall glycoproteins have been identified, including Gp70, an immunodominant antigen with opsonic capacity that enhances phagocytosis by macrophages and neutrophils [117]. These antibodies can modulate the inflammatory response and neutralize virulence factors. Likewise, immunization models with inactivated or purified *S. schenckii* antigens have shown that simultaneous activation of the Th1 and Th17 pathways confers partial protection against infection, accompanied by an increase in the production of IFN-γ, IL-17, and specific antibodies [119]. The generation of memory T cells and the persistence of high antibody titers suggest the possibility of inducing long-lasting protective immunity, which opens up prospects for the development of vaccines or immunotherapies based on fungal antigens [120].

Overall, the available evidence shows that the immune response to *S. schenckii* results from a dynamic interaction between the host’s innate and adaptive mechanisms. Early recognition of cell wall components such as β-glucans, glycans, PRM, and the Gp70 glycoprotein triggers the coordinated activation of macrophages, neutrophils, dendritic cells, and PBMCs, generating a proinflammatory microenvironment that favors the polarization of lymphocytes toward Th1 and Th17 phenotypes. These subpopulations, together with the production of specific antibodies, form an effective defense network capable of containing the infection and limiting its spread. However, *S. schenckii* has developed immune evasion strategies, including melanin production and cell wall reorganization, which allow it to reduce PAMP exposure, resist oxidative stress, and evade elimination by host effector cells.

## 5. Sporotrichosis Associated with *Sporothrix schenckii*

Sporotrichosis is a subcutaneous mycosis caused by members of the pathogenic clade of the *Sporothrix* genus and is reported in humans and other mammals, like cats. It is globally distributed with a high prevalence in tropical and subtropical areas, with several clinical forms, ranging from a fixed cutaneous form to a disseminated form that can affect deep-seated organs, such as bones and lungs [4,121]. The infection is commonly acquired by traumatic inoculation with contaminated vegetal matter (sapronosis) or the scratch-bite of an animal with sporotrichosis (zoonosis), with a high prevalence in immunocompetent patients [22]. Although *S. brasiliensis* is the species responsible for the highest number of cases currently recorded, *S. schenckii* continues to be a species of great clinical and epidemiological relevance, associated with numerous historical outbreaks and cases reported in different regions of the world. [122,123,124].

The main regions with *S. schenckii*-associated sporotrichosis are South Africa, Australia, North and Central America, Western South America, Madagascar, Thailand, Japan, and China [23,24,125]. This wide distribution reflects the cosmopolitan nature of the species and its ability to adapt to different ecological niches [4]. Throughout the 20^th^ century, *S. schenckii* was considered the classic etiological agent of sporotrichosis, responsible for multiple epidemic outbreaks, such as those in South Africa and the United States, associated with the handling of contaminated vegetables and decaying organic material [126]. These historical events laid the foundations for knowledge about the ecology, transmission, and clinical variability of the *Sporothrix* complex, consolidating *S. schenckii* as the most studied model within the genus [4].

Infections caused by *S. schenckii* show remarkable clinical variability, resulting from both the characteristics of the host and the biological diversity of the fungus itself [21]. In general terms, the fixed and lymphocutaneous skin forms are the most common, especially in immunocompetent individuals exposed through traumatic inoculation of contaminated plant material [12,21]. However, *S. schenckii* is also capable of causing extracutaneous manifestations, including osteoarticular, ocular, pulmonary, and disseminated forms, which can compromise deep organs and have a more severe course, particularly in immunocompromised patients [127,128]. These clinical variants reflect the fungus’s adaptive capacity to colonize different tissues, as well as its ability to modulate the host’s immune response and persist in adverse conditions. Taken together, the available evidence positions *S. schenckii* as a versatile pathogen with zoonotic and sapronotic potential, whose global distribution translates into a wide range of clinical presentations that vary in frequency and severity between geographic regions [129,130,131].

**Figure 3:**
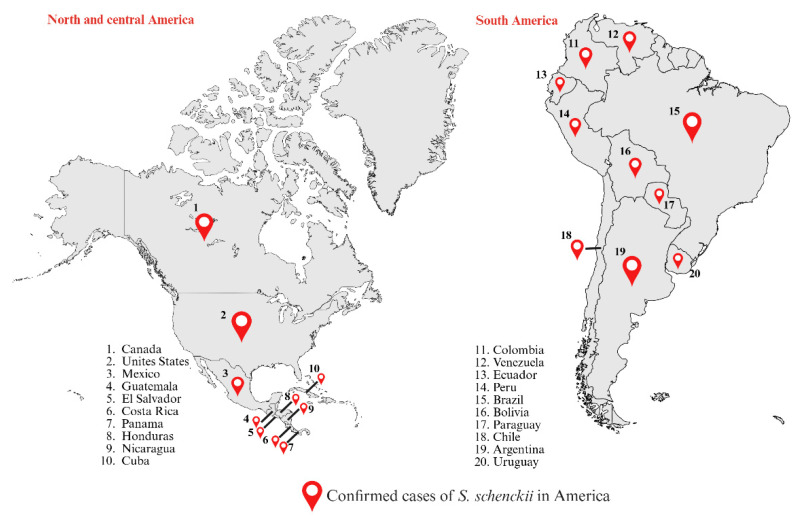
Geographic distribution of *Sporothrix schenckii* in North, Central, and South America. This species is predominantly distributed throughout the American continent. In North and Central America, *S. schenckii* has been reported in countries such as Canada, the United States, Mexico, Guatemala, El Salvador, Honduras, Nicaragua, Costa Rica, Cuba, and Panama. In South America, its presence has been documented in Colombia, Venezuela, Ecuador, Bolivia, Peru, Brazil, Paraguay, Uruguay, Argentina, and Chile.

In countries such as Canada, the United States, Mexico, Costa Rica, Guatemala, Honduras, Panama, Cuba, Venezuela, Colombia, Brazil, Peru, Paraguay, Uruguay, and Chile, numerous cases of sporotrichosis attributed to *S. schenckii* have been documented (Figure 3) [12,132,133,134,135,136]. Historical reports place the Americas as one of the regions with the greatest clinical and ecological diversity of this pathogen [4,12].

Molecular analyses have identified two main lineages: clade IIa, which includes homogeneous isolates from North and South America (Argentina, Bolivia, Colombia, and Peru), and clade IIb, restricted mainly to the Southern Cone, especially in Peru and Argentina, suggesting a continental expansion of the *S. schenckii* complex favored by environmental and occupational factors [4,137]. Several epidemic outbreaks have been documented in the United States during the 20^th^ century, one of the most significant being in 1988, when more than 80 people in 15 states were infected after handling contaminated sphagnum moss used in nurseries and gardening [138]. In Mexico, sporotrichosis is one of the most common subcutaneous mycoses, with more than 2700 cases recorded between 1914 and 2019, of which approximately 67% correspond to the lymphocutaneous form and 26% to the fixed form. Most patients were immunocompetent and acquired the infection through traumatic inoculation of contaminated plant material [1,121,139,140,141].

Extracutaneous forms, particularly osteoarticular and pulmonary, have also been described, accounting for about 10–15% of cases, mainly in individuals with predisposing factors, such as alcoholism or immunosuppression [12,127,128]. In Central America, countries such as Costa Rica, Guatemala, and Panama have reported sporadic cases related to the handling of contaminated soil and vegetation, while in western South America, *S. schenckii* remains the main etiological agent of human sporotrichosis in regions of Venezuela, Colombia, Peru, and Chile [4,133,136]. Overall, epidemiological evidence shows that more than 80% of *S. schenckii* infections in the Americas are cutaneous (fixed or lymphocutaneous), while systemic manifestations constitute a smaller but clinically relevant percentage, reflecting the fungus’s adaptive capacity to colonize different tissues and persist under diverse immunological conditions [4].

**Figure 4:**
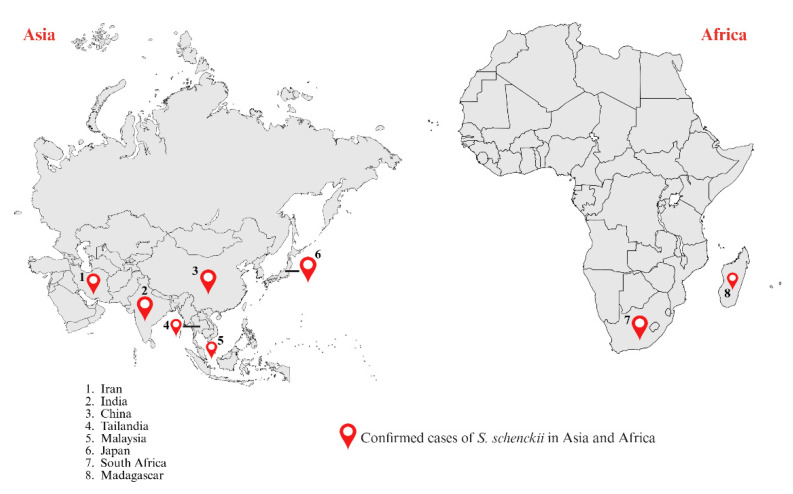
Geographic distribution of *Sporothrix schenckii* in Asia and Africa. This species is widely distributed in Asia, with cases reported in China, Japan, India, Thailand, Malaysia, and Iran, involving both sapronotic and zoonotic transmission. In Africa, *S. schenckii* has been historically associated with major outbreaks in South Africa and sporadic cases in Madagascar, highlighting its persistence in environmental and occupational settings.

In Asia, *S. schenckii* continues to be the main species associated with human sporotrichosis, with widespread distribution in countries such as China, Japan, India, and Thailand (Figure 4) [142,143,144]. In China, this mycosis is one of the most prevalent subcutaneous infections, with more than 3000 cases documented in recent decades, of which more than 90% are attributed to *S. schenckii* [4,122,145]. The northeastern provinces, particularly Jilin, Heilongjiang, and Liaoning, have the highest disease burden, favored by the cold and humid climate that promotes the survival of the fungus in the soil and in decaying plant matter [4,122]. Most infections are cutaneous, especially the fixed and lymphocutaneous forms in immunocompetent individuals. However, extracutaneous osteoarticular, pulmonary, and ocular cases have been reported, especially in immunocompromised patients or those with a history of prolonged corticosteroid treatment [122,146]. In Japan, *S. schenckii* has historically been the predominant agent, with numerous cases reported in agricultural workers, gardeners, and flower growers exposed to contaminated organic material [4]. Cases are mainly concentrated in temperate regions, where humidity and intensive agricultural activities favor transmission; in addition, a marked seasonality has been observed, with peaks in incidence in spring and early summer [4].

In India, *S. schenckii* has been recognized as the predominant etiological agent since the mid-20^th^ century, with most cases concentrated in the northern and northeastern states, particularly Himachal Pradesh, Uttarakhand, and Sikkim [4]. Between 1960 and 2013, more than 400 cases were documented, with marked endemicity in mountainous regions with a temperate and humid climate, where the fungus is associated with agricultural soils and decaying vegetation. The lymphocutaneous form accounts for about 75–80% of infections, followed by the fixed form. However, extracutaneous forms, mainly osteoarticular and pulmonary, have been described in immunocompromised patients or those undergoing prolonged steroid treatment [147]. Localized outbreaks have been linked to rural activities, such as handling hay and thorny branches, while in urban settings, cases related to gardening have been reported.

In Thailand, sporadic cases of sporotrichosis have been reported through both sapronotic and zoonotic transmission. In one of the first reports, a 71-year-old man with chronic diseases and owner of an infected cat developed progressive lymphocutaneous lesions after being scratched, while a healthy 31-year-old woman developed skin ulcers after contact with a sick cat. In both cases, *S. schenckii* was identified by culture and mass spectrometry [4,148]. Recent studies confirm the presence of *S. schenckii* in Thai human isolates, suggesting the persistence of endemic foci in tropical rural areas, as well as the possibility of transmission between domestic animals and humans [143].

In Malaysia, a six-year study recorded 19 confirmed cases, of which 68.4% were lymphocutaneous, and a similar proportion were patients with a history of trauma or cat bites [143]. Subsequently, molecular analysis identified 25 clinical isolates of *S. schenckii* in humans and cats, confirming that all were *S. schenckii* and shared genetic identity, suggesting the presence of a clonal lineage in the country. The coexistence of human and feline strains reinforces the relevance of zoonotic transmission, in addition to the traditional sapronotic route [149,150]. In addition, in other countries, such as Iran, eight cases of sporotrichosis were reported, five women and three men between the ages of 23 and 60, with a predominance of fixed and lymphocutaneous forms, and one case of osteoarticular sporotrichosis [94]. All isolates corresponded to *S. schenckii*, with no evidence of zoonotic transmission, suggesting that infection in that region persists mainly as a sapronosis of environmental origin.

On the African continent, *S. schenckii* has historical significance as the classic agent of sporotrichosis [151]. South Africa was the scene of some of the most extensive outbreaks recorded worldwide, especially between the 1940s and 1980s, when thousands of gold miners contracted the infection after coming into contact with contaminated wood used in tunnel construction [126,151]. During this period, it was estimated that more than 3000 cases occurred in the Transvaal region, consolidating South Africa as a historical endemic focus of the disease. The predominant clinical manifestations were the lymphocutaneous and fixed forms, while extracutaneous presentations were rare and mainly observed in immunocompromised individuals. Although the incidence has declined since the end of occupational outbreaks, sporotrichosis remains endemic in some rural regions of South Africa and Madagascar, where *S. schenckii* continues to be the most frequent etiological agent (Figure 4) [4,23].

In contrast to endemic regions in the Americas, Asia, and Africa, sporotrichosis is considered an uncommon mycosis in Europe [152,153,154]. Most reported cases correspond to sporadic autochthonous infections or are associated with travel to endemic areas. Nevertheless, several cases caused predominantly by *S. schenckii* have been documented in European countries, such as Spain, Italy, France, and the United Kingdom [155,156,157]. The low prevalence of sporotrichosis in Europe has been attributed to climatic conditions less favorable for the environmental persistence of *Sporothrix* species, as well as differences in occupational exposure and ecological niches [156].

More recently and unusually, cases of osteoarticular sporotrichosis caused by *S. schenckii* have been reported, characterized by a chronic and indolent course that often delays diagnosis and initiation of appropriate treatment. These infections usually involve large joints, such as the knee, elbow, or ankle, with nonspecific clinical findings that mimic bacterial or mycobacterial arthritis [131,158,159,160]. In most cases, the infection originates from hematogenous spread or direct extension from a previous skin lesion, although primary cases with no apparent entry point have also been documented [132]. Vertebral, synovial, and tenosynovial infections have also been described in immunocompromised patients, chronic alcoholics, or patients undergoing prolonged treatment with corticosteroids [127]. The clinical course is usually prolonged, with periods of months or even years before definitive diagnosis, and isolation of the fungus in synovial fluid or bone biopsies remains the gold standard for etiological confirmation.

Overall, global epidemiological evidence shows that *S. schenckii* maintains a wide geographical distribution and remarkable ecological plasticity, allowing it to adapt to diverse environments and hosts. Although transmission patterns differ between regions, with sapronosis predominating in Asia and Africa and sapronotic and zoonotic routes coexisting in the Americas, the species retains a remarkable capacity for environmental persistence and infection in immunocompetent individuals. The cutaneous, fixed, and lymphocutaneous clinical forms continue to be the most common forms in all continents. However, extracutaneous cases, although less common, represent a diagnostic and therapeutic challenge, especially in patients with immunosuppression or underlying chronic diseases. This overview highlights the biological and pathogenic versatility of *S. schenckii*, as well as the need to strengthen epidemiological surveillance and molecular studies to better understand the transmission dynamics and genetic diversity of this species globally.

### Domestic Animals’ Infection by Sporothrix schenckii

*S. schenckii* infections in domestic animals represent an important, although often underestimated, route of transmission to humans [161]. Even though most of the literature on *Sporothrix* zoonosis has focused on *S. brasiliensis*, especially in cats in Brazil, *S. schenckii* continues to be reported in cats, dogs, and other domestic mammals in different countries [4,123,124].

Young adult cats, males, with uncontrolled outdoor access are those most frequently stricken by sporotrichosis, associated with inter-animal aggression [162,163]. The clinical presentation can range from isolated cutaneous lesions to fatal systemic ones [21]. In the first case, the most common lesion pattern is cutaneous ulcers, granulomatous nodules, and crusts, as well as lymphangitis and lymphadenitis on different parts of the body, especially on the head, nose, ears, distal limbs, and base of the tail [164,165]. The most frequently documented extracutaneous manifestations are associated with respiratory involvement, including symptoms such as sneezing, rhinorrhea, and dyspnea. These are directly linked to therapeutic failure and increased mortality [166,167]. Mucous membrane involvement, notably affecting the ocular mucosa, is also commonly reported in feline cases [164,168]. Notably, concurrent infections with feline leukemia virus or feline immunodeficiency virus do not significantly influence the clinical presentation or prognosis in affected cats [21,169].

Feline sporotrichosis caused by *S. schenckii* has been documented in Europe, the United States, and Asia. However, the disease has not exhibited the same clinical or epidemiological impact as the hyperendemic situation associated with *S. brasiliensis* in Brazil [143,170,171,172,173]. Sporotrichosis caused by *S. schenckii* in Japan, Malaysia, and Thailand has been observed with a clinical pattern characterized by a high prevalence of lesions located in cooler areas of the feline body, such as the nasal region and especially the tips of the ears [163,172,174]. This distribution could be related to the lower relative heat tolerance of *S. schenckii* compared to *S. brasiliensis*, which would favor its growth in peripheral areas of the feline body, where the temperature is lower than in the core. Given that cats have an average physiological temperature of 38–39 °C, this difference could influence the location of lesions and susceptibility to deeper infections.

In a recent study, the in vitro interaction between *S. schenckii* and feline phagocytes was evaluated, observing a lower rate of phagocytosis and cytotoxicity compared to *S. brasiliensis*, as well as morphophysiological differences, including early hyphal formation, suggesting specific mechanisms of immune evasion and persistence [118]. In Australia, although cases of feline sporotrichosis are rare, a high fungal load has been reported in skin lesions, representing a significant risk of zoonotic transmission [108].

A classic study conducted in Brazil isolated *S. schenckii* in 100% of 148 cats with clinical signs and in 66.2% of nasal cavities, 41.8% of oral cavities, and 39.5% of nails, even in some apparently healthy animals in household contact with positive cases. These findings suggest the possibility of asymptomatic colonization and its role as a domestic reservoir [161]. Similarly, a more recent report in Belém (Pará, Brazil) documented the first isolation of *S. schenckii* in a cat with possible zoonotic transmission to its owner, highlighting the underreporting of these cases and the persistent risk in urban environments [175].

**Figure 5:**
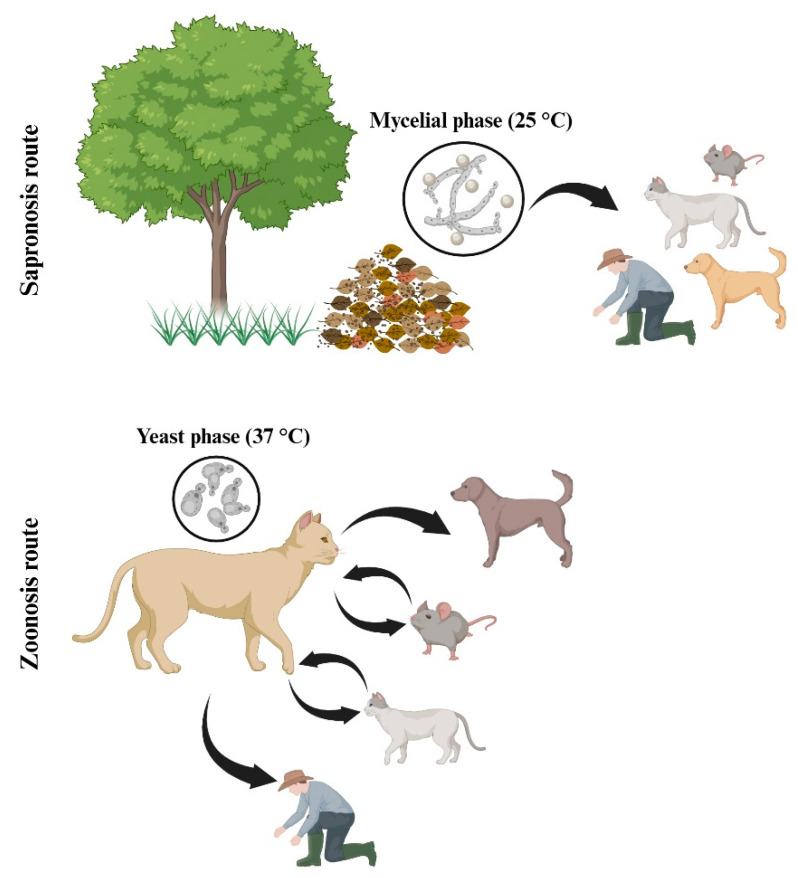
Transmission routes of *Sporothrix schenckii*. The sapronosis route originates from the mycelial phase (25 °C) present in soil, decaying vegetation, and plant material, where conidia can be inoculated into humans or animals through traumatic injury. In the host, the fungus undergoes a dimorphic transition to the yeast phase, initiating infection. The zoonotic route mainly involves transmission from infected cats to humans, dogs, or other mammals through scratches, bites, or contact with exudates from ulcerated lesions. The zoonotic route mainly involves transmission from infected cats to humans, dogs, or other mammals through scratches, bites, or contact with exudates from ulcerated lesions. In addition, cats can acquire the infection from mice, and transmission can also occur in the opposite direction.

Overall, the available evidence indicates that cats, particularly unneutered males with free access to the outdoors and prone to fighting or biting, act as reservoirs and sources of transmission of the fungus (Figure 5). Although most recent studies focus on *S. brasiliensis*, the clinical and epidemiological relevance of *S. schenckii* in the zoonotic cycle persists. These findings underscore the need to strengthen veterinary surveillance, environmental control, and health education.

Dogs are the second most affected pets, mainly because of their close contact with cats. *S. brasiliensis* and *S. schenckii* are the primary species causing clinical sporotrichosis in this vertebrate (Figure 5) [163,176,177,178]. However, in dogs, the condition occurs less frequently and is often characterized by multiple cutaneous and subcutaneous lesions on the head, ears, and thorax. Osteoarticular and disseminated manifestations have also been documented, though they are rare [176,178]. Transmission can result from injuries incurred during hunting activities, such as those caused by thorns or wood splinters [177].

A recent case reported in Brazil described a natural infection by *S. schenckii* in a guinea pig (*Cavia porcellus*), which is the first documented case in this species [179]. The animal, which lived with five cats with free access to the outdoors, showed exudative crusted lesions on the dorsal region, with the infection confirmed by PCR and direct cytology. Treatment with itraconazole (5 mg/kg/day for 45 days), combined with the topical application of ozonated sunflower oil, achieved complete remission of the lesions [179]. This finding broadens the spectrum of hosts susceptible to the *S. schenckii* complex and reinforces the role of cats as the primary source of transmission to other domestic animals and humans.

Taken together, these cases confirm that *S. schenckii* maintains a broad adaptive capacity among domestic and companion mammals, including canines and rodents, and that interspecies transmission occurs frequently in environments where infected animals coexist. The identification of new host species, such as guinea pigs, highlights the importance of active veterinary surveillance, early molecular diagnosis, and the implementation of biosecurity measures in the management of infected animals.

## 6. Identification and Diagnostic

Detection and diagnosis of sporotrichosis caused by *S. schenckii* represents a challenge; there are reports about clinical cases where the diagnosis is not correct and, as a consequence, neither the treatment [180].

**Figure 6:**
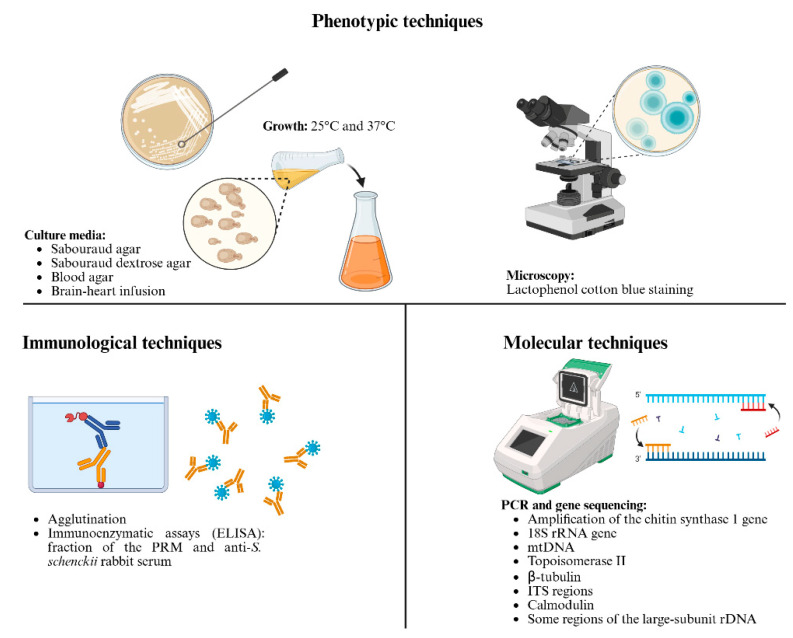
Schematic overview of the main techniques currently used for the identification and diagnosis of *Sporothrix schenckii*. Phenotypic methods include culturing in different media at 25 °C and 37 °C, and microscopic examination using lactophenol cotton blue staining to evaluate characteristic morphological features. Immunological techniques are based on agglutination and immunoenzymatic assays (ELISA) using PRM fractions and anti-*S. schenckii* sera. Molecular approaches comprise PCR-based methods and gene sequencing targeting conserved and taxonomically informative loci, including *CHS1*, 18S rRNA, mtDNA, topoisomerase II, β-tubulin, ITS regions, calmodulin, and selected regions of the large-subunit rDNA.

First-line phenotypic techniques, such as cultures in different media like Sabouraud agar, Sabouraud dextrose agar, blood agar, and brain-heart infusion at different temperatures (25 °C and 37 °C), are commonly used in the identification of *S. schenckii*. These techniques allow for observing the typical forms and color of the colonies between 5 and 10 days, as well as the characteristic mycelium and yeast-like cell morphologies when inspected under the microscope [143,180]. Lactophenol cotton blue staining is also used as a confirmatory technique due to its effect on the fungal structures; in the case of *S. schenckii* is easy to observe the typical septate hypha and the conidia flower bouquet [143] (see Figure 6).

On the other hand, immunological techniques have been described for the identification of *S. schenckii*, based on agglutination and immunoenzymatic assays that were performed with a fraction of the PRM and anti-*S. schenckii* rabbit serum (Figure 6). This cell wall fraction was recognized by patients’ IgG antibodies; however, it is not effective in the diagnosis of patients with acute sporotrichosis or with past infections. Furthermore, the identification at the species level is not possible [181]. Thus, more research is necessary on this kind of technique to be useful in the future.

Although the techniques mentioned are very helpful in the correct diagnosis, it is necessary the use more precise tools for the confirmation of an infection caused by *Sporothrix* species. For *S. schenckii*, molecular tools have been reported to be useful in their identification. The amplification of the chitin synthase 1 gene by polymerase chain reaction (PCR) was the first molecular technique described in the identification of this fungus [182]. Another PCR assay has been designed to target the 18S rRNA gene sequence, used as a confirmatory method to isolate previously evaluated by phenotypic techniques, helping in a fast a correct diagnosis in the sporotrichosis cases [183]. In Mexico, a technique based in the restriction fragment length polymorphism of the mitochondrial DNA was used to characterize thirteen *S. schenckii* isolates coming from different sporotrichosis cases, finding out that all of them belonged to the group A in a phylogenic tree previously established for the mitochondrial DNA, confirming the prevalence of the group A in the Americas and the differences between these isolates [184]. In the same way, other targets as the topoisomerase II gene, β-tubulin gene, ITS regions, or some regions of the large-subunit rDNA, like D1-D2 in the 28S, have been used for the identification of genetic differences between strains of the same species or for the first identification of *S. schenckii* in some regions that have not been identified before [153]. Sequence analysis of the calmodulin gene and the use of the universal primer T3B in PCR fingerprinting have been reported as useful in the differentiation of species like *S. schenckii* from other closely related species, providing valuable information about the variations that could exist even between members of the same species, opening the possibilities at the moment of confirm a microbiological data in combination with one or more molecular techniques, making easier a fast and correct identification of *S. schenckii* [185] (Figure 6).

Recently, a new method based on a multiplex real-time PCR using as target the calmodulin gene showed a high fidelity at the moment of the identification between different *Sporothrix* species, being able to detect a coinfection or giving a positive result in strains that were not able to grow in a typical culture. This could represent a new alternative that does not need previous phenotypic results, saving time and giving the patient the possibility of start its treatment as soon as possible. However, it is mentioned that this method has not been tested with clinical isolates, and it is necessary to prove its efficacy in the field [186].

Matrix-assisted laser-desorption/ionization time-of-flight mass spectrometry is another technique that has been used to identify species in a fast way, based on the analysis of the ribosomal proteins, and has demonstrated efficacy at the moment of being compared with other techniques, such as the calmodulin gene sequencing. The work made by Oliveira et al. [187] showed a validation of a method that allowed the identification of different species of *Sporothrix* spp., and at the same time, the enrichment of a database library to be used as a guide in the future identification of species like *S. schenckii* [187]. This technique has already been used in the identification of *S. schenckii* in clinical cases of patients with sporotrichosis caused by this pathogen [143], confirming its efficacy in combination with other identification methods.

## 7. Treatment

The therapeutic management of sporotrichosis depends primarily on the clinical form, the patient’s immune status, and the antifungal susceptibility of the isolate. Over time, treatment strategies have evolved from the use of iodinated compounds to second-generation azoles, which currently represent the basis of management with excellent clinical results [21,157].

Itraconazole remains the drug of choice for most cutaneous and lymphocutaneous forms [21,188]. This antifungal agent is effective and safe, with low toxicity and good tolerance even in prolonged treatments [189]. It is administered orally at doses of 100–200 mg/day for an average period of 3 to 6 months, extending therapy for at least 2 to 4 weeks after complete clinical resolution of the lesions [21,25,157]. Its effectiveness is related to its adequate oral bioavailability and fungistatic activity against *S. schenckii*, with minimum inhibitory concentrations (MICs) reported between 0.03 and 1 µg/mL [190]. In cases of disseminated or refractory disease, the dose may be increased to 200 mg every 12 h.

Terbinafine represents a safe and effective therapeutic alternative, with in vitro activity comparable to that of itraconazole and MICs ranging from 0.03 to 0.5 µg/mL [191]. It is administered at doses of 250 to 1000 mg/day, with a treatment duration similar to that of itraconazole [192]. In a comparative study, doses of 250 mg/day of terbinafine and 100 mg/day of itraconazole achieved cure rates of 92.7% and 92%, respectively, demonstrating that terbinafine is an equally effective and well-tolerated option for cutaneous sporotrichosis [193]. Clinical studies have also shown that the combination of terbinafine and itraconazole can have a synergistic effect, shortening the time to resolution of lesions [21]. However, the high cost of terbinafine compared to itraconazole remains a significant limitation, especially in developing countries [188].

Another alternative treatment for uncomplicated cutaneous forms is potassium iodide, one of the oldest treatments, which continues to be an effective and economical alternative, especially in rural areas or areas with limited access to azole antifungals. However, its use has been limited by adverse gastrointestinal effects, hypersalivation, thyroid disorders, and low tolerability [194,195].

Disseminated, osteoarticular, pulmonary, or meningeal forms of sporotrichosis require a more aggressive therapeutic approach [1,25]. In these cases, amphotericin B is the treatment of choice, especially in patients with systemic involvement or immunosuppression. The liposomal formulation (3–5 mg/kg/day) is recommended due to its better safety profile and lower nephrotoxicity. However, when it is not available, the deoxycholate formulation (0.7–1 mg/kg/day) can be used until clinical improvement is achieved [188]. Once the initial response has been achieved, consolidation therapy with itraconazole (200 mg every 12 h) is indicated for a period of 6 to 12 months to prevent relapse and ensure complete resolution of the infection [188]. In cases of meningeal or disseminated disease, it may be necessary to prolong antifungal therapy or maintain a long-term suppressive therapy regimen [16]. Lipid formulations of amphotericin B have demonstrated comparable efficacy with a significant reduction in renal toxicity, making them the preferred option when available.

In this context, second-generation azoles, such as posaconazole and voriconazole, have shown excellent in vitro activity against the *S. schenckii* complex, with average MICs between 0.06 and 0.5 µg/mL, although clinical evidence remains limited [196,197]. A recent comparative study evaluated the susceptibility of various species of the genus *Sporothrix* to conventional and new-generation agents, demonstrating greater sensitivity of *S. schenckii* to posaconazole and voriconazole, while some strains showed a lower response to fluconazole and amphotericin B, reinforcing the importance of antifungal monitoring and targeted therapy [198]. In contrast, fluconazole has inconsistent activity and is not recommended as a first-line drug [188]. In recent years, combination therapeutic strategies have been explored, such as itraconazole–terbinafine or amphotericin B–azoles, which have shown promising results both in vitro and in refractory clinical cases, suggesting a possible synergistic effect [21,193].

Likewise, there has been growing interest in natural metabolites with antifungal potential, including terpenoids, flavonoids, and phenolic compounds, which have demonstrated inhibitory activity against *S. schenckii* and could act as adjuvants in conventional therapeutic regimens [199]. Although these alternatives are still in the experimental stages, their study broadens the prospects for the development of new antifungal agents with a better safety profile and lower risk of resistance [21].

Although conventional antifungal therapies and new combination strategies have significantly improved the prognosis of sporotrichosis, clinical management remains challenging in immunocompromised patients [200,201]. In individuals with HIV infection, undergoing immunosuppressive treatment, or with severe comorbidities, *S. schenckii* infections tend to have a longer, more refractory course and a higher risk of systemic dissemination. In these cases, antifungal treatment should be maintained for longer periods, accompanied by close clinical and mycological monitoring, as well as correction of immunosuppression when possible [200].

At the same time, complementary immunotherapeutic strategies have been explored to enhance the host response [202,203]. In murine models, immunization with cell wall proteins and the use of monoclonal antibodies directed against the Gp70 glycoprotein, a surface adhesin of *S. schenckii*, have been shown to reduce the fungal load and modulate the inflammatory response, opening up new perspectives for the integration of immunomodulatory approaches in the treatment of sporotrichosis [202].

In special populations, treatment must be carefully adjusted. In pregnant women, amphotericin B is the drug of choice, given the teratogenic risk associated with the use of azoles and the adverse thyroid effect of potassium iodide [188]. In pediatric patients, both itraconazole and potassium iodide are safe and effective, with doses adjusted to body weight and a favorable clinical response [21,188].

Overall, therapeutic advances have substantially improved the prognosis of sporotrichosis caused by *S. schenckii*. However, the recent identification of strains with reduced susceptibility to itraconazole and amphotericin B [198], variability in clinical response among species of the *S. schenckii* complex, and the absence of specific therapies for severe or refractory forms represent persistent challenges. These limitations underscore the need to develop new antifungal compounds, optimize combination strategies, and advance immunotherapeutic approaches that improve the efficacy and reduce the toxicity of available treatments [204]. A summary of the main antifungal agents, their indications, and dosage is provided in Table 3.

## 8. Conclusions

*S. schenckii* remains one of the most representative and versatile species within the *Sporothrix* pathogenic clade, notable for its wide geographical distribution, remarkable phenotypic plasticity, and ability to infect multiple hosts. Although recent scientific attention has focused on *S. brasiliensis*, *S. schenckii* continues to play an essential role in understanding the evolutionary, physiological, and molecular mechanisms underlying sporotrichosis and other dimorphic mycoses.

Advances in genomics, transcriptomics, and proteomics have made it possible to delineate the main components involved in virulence and host adaptation, including factors associated with cell wall remodeling, dimorphic transition, melanin production, and adhesin expression. However, many of the mechanisms that regulate these responses remain unclear, and their elucidation could open new avenues for the design of targeted therapies and more effective immunomodulatory strategies.

In the future, it will be a priority to conduct more in-depth comparative studies between species of the *S. schenckii* complex, with special attention to differences in virulence, immune response, antifungal susceptibility, and zoonotic transmission capacity.

Overall, *S. schenckii* remains a reference species in modern medical mycology. A comprehensive study covering everything from molecular biology to ecological and clinical dynamics will provide the basis for developing more sensitive diagnostic tools, safer therapies, and preventive strategies adapted to current epidemiological realities. Consolidating this knowledge will strengthen our understanding of emerging mycoses and contribute significantly to global public health.

## Figures and Tables

**Table 1:** Prediction of some important virulence factors and determinants in *Sporothrix schenckii*.

Virulence Factors	Organism	Protein	*S. schenckii* (Locus Tag)	E-Value *	Similarity (%) *
Adhesins	*Candida albicans*	Als 1-9	No found	-	-
		Eap1	No found	-	-
		Ecm33	SPSK_05317	6 × 10^−46^	73
		Hwp1	No found	-	-
		Iff4	No found	-	-
		Int1	SPSK_07346	8 × 10^−52^	50
		Mp65	SPSK_05120	1 × 10^−38^	74
	*Aspergillus fumigatus*	RodA RodB	No found	-	-
		AspF2	No found	-	-
		CalA	SPSK_05470	2 × 10^−94^	71
		Scw11	SPSK_04001	3 × 10^−137^	69
		Gel1	SPSK_05276	0	70
		Gel2	SPSK_04169	2 × 10^−159^	99
		Mp1	No found	-	-
		AfCalAp	No found	-	-
	*Cryptococcus neoformans*	Cfl1	No found	-	-
		Cpl1	No found	-	-
		Mp98	SPSK_03393	2 × 10^−24^	44
Biofilm	*C. albicans*	Bcr1	SPSK_01505	2 × 10^−24^	76
		Brg1	SPSK_05129	1 × 10^−13^	78
		Efg1	SPSK_07078	1 × 10 ^−57^	65
		Hsp90	SPSK_08698	0	85
		Ndt80	SPSK_09140	3 × 10^−9^	37
		Rob1	SPSK_03010	7 × 10^−8^	54
		Csr1	SPSK_08605	1 × 10^−33^	57
	*C. neoformans*	Lac1	SPSK_03091	3 × 10^−24^	60
		Ure1	SPSK_00695	0	99
		Cap59	SPSK_09241	5 × 10^−15^	50
Hydrolytic enzymes	*C. albicans*	Lip5-8	SPSK_03375	1 × 10^−60^	86
		Sap1-8	SPSK_06273	2 × 10^−52^	53
		Plb1-3	SPSK_01063	4 × 10^−145^	57
	*A. fumigatus*	Pep1	SPSK_02149	0	60
		Pep2	SPSK_00526	0	85
		Ap1	SPSK_07865	8 × 10^−94^	97
		CtsD	SPSK_01559	6 × 10^−82^	58
		PlaA	SPSK_02253	2 × 10^−136^	50
Dimorphism	*C. albicans*	Cph1	SPSK_07311	3 × 10^−72^	78
		Hgc1	SPSK_05321	4 × 10 ^−21^	42
		Nrg1	SPSK_00519	1 × 10 ^−10^	55
		Tup1	SPSK_02314	1 × 10 ^−139^	67
	*C. neoformans*	Mob2	SPSK_01925	9 × 10 ^−44^	57
		Cbk1	SPSK_06025	1 × 10 ^−178^	68
		Tao3	SPSK_02910	4 × 10 ^−129^	44
		Sog2	SPSK_03988	9 × 10 ^−113^	51
Thermotolerance	*C. albicans*	Hsp60	SPSK_01586	0	87
		Hsp104	SPSK_08586	0	65
		Ssa1	SPSK_08625	0	88
		Ssb1	SPSK_03121	0	87
	*A. fumigatus*	CrgA	SPSK_09995	6 × 10 ^−42^	79
		Sch9	SPSK_10850	0	71
		Hsf1	SPSK_08498	7 × 10 ^−96^	48
		BiP/Kar2	SPSK_04019	0	87
		Ssc70	SPSK_03148	0	88
		Hsp88	SPSK_00430	0	75
		BiP	SPSK_06078	0	69
		Lhs1/Orp150	SPSK_02198	0	61
		Hsp90	SPSK_08698	0	91
	*C. neoformans*	Ccr4	SPSK_07136	2 × 10 ^−141^	53
Immune evasion	*C. albicans*	Hgt1	SPSK_06192	7 × 10 ^−116^	
		Hmx1	No found	-	-
		Msb2	SPSK_07127	9 × 10 ^−18^	44
		Pra1	No found	-	-
		Rbt5	No found	-	-
		Sit1	SPSK_02970	5 × 10 ^−150^	64
	*A. fumigatus*	Hyp1/RodA	No found	-	-
		Pksp/Alb1	SPSK_00653	0	60
	*C. neoformans*	Rim101	SPSK_07198	2 × 10 ^−36^	70
Melanin production	*A. fumigatus*	Fet3	SPSK_07279	0	68
		TilA	SPSK_04101	6 × 10 ^−168^	62
		Dihydrogeodin/ laccase	SPSK_07219	2 × 10 ^−99^	46
Cell wall synthesis	*C. albicans*	Fks1	SPSK_01365	2 × 10 ^−79^	78
		Dpm3	SPSK_02816	2 × 10 ^−19^	63
		Pmt2	SPSK_08548	0	65
	*A. fumigatus*	ChsG	SPSK_06989	0	76
		ChsA	SPSK_08492	1 × 10 ^−112^	88
		ChsF	SPSK_04841	2 × 10 ^−74^	95
		Dpm2	SPSK_08145	2 × 10 ^−32^	83
		Pmt1	SPSK_05892	0	72
		Pmt4	SPSK_08628	0	78
		Kre2/Mnt1	SPSK_09069	0	88
		Ktr4	SPSK_05332	0	74
		Och1	SPSK_03245	1 × 10 ^−37^	51
		Mnn9	SPSK_09403	9 × 10 ^−158^	75

Protein nomenclature corresponds to accession codes of the National Center for Biotechnology Information database (https://www.ncbi.nlm.nih.gov/; accessed on August 2025). Protein sequences from *Candida albicans*, *Aspergillus fumigatus*, and *Cryptococcus neoformans* were used as queries for BLASTp analysis using the standard BLASTp algorithm (https://blast.ncbi.nlm.nih.gov/Blast.cgi?PAGE=Proteins, accessed on 15 August 2025). Searches were performed against the annotated *Sporothrix schenckii* protein database derived from the: *Sporothrix schenckii* (taxid:29908) genome available at NCBI. The best hit was reported in the *S. schenckii* column. * The E-value and similarity percentages refer to pairwise amino acid sequence comparisons between each query protein and the corresponding putative *S. schenckii* ortholog.

**Table 2:** Virulence factors reported to *S. schenckii*.

Virulence Factors	Protein	Reference
Adhesins	Gp70	[35,50]
	Hsp60	[29]
	Pap1	[29]
Proteases	Proteinase I	[52]
	Proteinase II	[52]
Thermotolerance	Hsp90	[26]
	Sscmk1	[53]

**Table 3:** Antifungal agents currently used in the treatment of sporotrichosis caused by *Sporothrix schenckii*.

Antifungal Agent	Clinical Indication	Dose and Duration	References
Itraconazole	Cutaneous and lymphocutaneous sporotrichosis	100–200 mg/day for 3–6 months; extend 2–4 weeks after clinical resolution	[21,25,182,183,184,185]
Terbinafine	Alternative for cutaneous forms	250–1000 mg/day for 3–6 months	[186,187,188]
Amphotericin B (liposomal)	Disseminated, severe, or immunocompromised cases	3–5 mg/kg/day, followed by itraconazole consolidation	[1,25,183]
Amphotericin B (deoxycholate)	Severe disease when liposomal formulation is unavailable	0.7–1 mg/kg/day until improvement	[183]
Posaconazole/Voriconazole	Refractory cases or intolerance to first-line agents	Variable; case-dependent	[191,192,193]
Potassium iodide	Uncomplicated cutaneous sporotrichosis	Gradual dose escalation until clinical response	[189,190]

## Data Availability

The original contributions presented in the study are included in the article, further inquiries can be directed to the corresponding authors.

## References

[1] Lopes-Bezerra L.M., Mora-Montes H.M., Bonifaz A., Mora-Montes H.M., Lopes-Bezerra L.M. (2017). *Sporothrix* and sporotrichosis. Current Progress in Medical Mycology.

[2] López-Romero E., Reyes-Montes M.d.R., Perez-Torres A., Ruiz-Baca E., Villagomez-Castro J.C., Mora-Montes H.M., Flores-Carreon A., Toriello C. (2011). *Sporothrix schenckii* complex and sporotrichosis, an emerging health problem. Future Microbiol..

[3] de Beer Z.W., Duong T.A., Wingfield M.J. (2016). The divorce of *Sporothrix* and *Ophiostoma*: Solution to a problematic relationship. Stud. Mycol..

[4] Chakrabarti A., Bonifaz A., Gutierrez-Galhardo M.C., Mochizuki T., Li S. (2015). Global epidemiology of sporotrichosis. Med. Mycol..

[5] Mora-Montes H.M., Dantas Ada S., Trujillo-Esquivel E., de Souza Baptista A.R., Lopes-Bezerra L.M. (2015). Current progress in the biology of members of the *Sporothrix schenckii* complex following the genomic era. FEMS Yeast Res..

[6] Etchecopaz A.N., Lanza N., Toscanini M.A., Devoto T.B., Pola S.J., Daneri G.L., Iovannitti C.A., Cuestas M.L. (2020). Sporotrichosis caused by Sporothrix brasiliensis in Argentina: Case report, molecular identification and in vitro susceptibility pattern to antifungal drugs. J. Mycol. Med..

[7] Xavier M.O., Poester V.R., Trápaga M.R., Stevens D.A. (2023). *Sporothrix brasiliensis*: Epidemiology, therapy, and recent developments. J. Fungi.

[8] Mora-Montes H.M. (2022). Special Issue “*Sporothrix* and Sporotrichosis 2.0”. J. Fungi.

[9] Gremião I.D.F., Martins da Silva da Rocha E., Montenegro H., Carneiro A.J.B., Xavier M.O., de Farias M.R., Monti F., Mansho W., de Macedo Assunção Pereira R.H., Pereira S.A. (2021). Guideline for the management of feline sporotrichosis caused by *Sporothrix brasiliensis* and literature revision. Braz. J. Microbiol..

[10] Brown G.D., Denning D.W., Gow N.A., Levitz S.M., Netea M.G., White T.C. (2012). Hidden killers: Human fungal infections. Sci. Transl. Med..

[11] Mora-Montes H.M. (2018). Special Issue “*Sporothrix* and Sporotrichosis”. J. Fungi.

[12] Hernández-Castro R., Pinto-Almazán R., Arenas R., Sánchez-Cárdenas C.D., Espinosa-Hernández V.M., Sierra-Maeda K.Y., Conde-Cuevas E., Juárez-Durán E.R., Xicohtencatl-Cortes J., Carrillo-Casas E.M. (2022). Epidemiology of clinical sporotrichosis in the Americas in the last ten years. J. Fungi.

[13] Téllez M.D., Batista-Duharte A., Portuondo D., Quinello C., Bonne-Hernández R., Carlos I.Z. (2014). *Sporothrix schenckii* complex biology: Environment and fungal pathogenicity. Microbiology.

[14] Marimon R., Cano J., Gené J., Sutton D.A., Kawasaki M., Guarro J. (2007). *Sporothrix brasiliensis*, *S. globosa*, and *S. mexicana*, three new *Sporothrix* species of clinical interest. J. Clin. Microbiol..

[15] de Lima Barros M.B., Schubach A.O., de-Vasconcellos Carvalhaes De-Oliveira R., Martins E.B., Teixeira J.L., Wanke B. (2011). Treatment of cutaneous sporotrichosis with Itraconazole—Study of 645 patients. Clin. Infect. Dis..

[16] Orofino-Costa R., Macedo P.M., Rodrigues A.M., Bernardes-Engemann A.R. (2017). Sporotrichosis: An update on epidemiology, etiopathogenesis, laboratory and clinical therapeutics. An. Bras. Dermatol..

[17] Rodrigues A.M., de Hoog G.S., de Cássia Pires D., Brihante R.S., Sidrim J.J., Gadelha M.F., Colombo A.L., de Camargo Z.P. (2014). Genetic diversity and antifungal susceptibility profiles in causative agents of sporotrichosis. BMC Infect. Dis..

[18] Tamez-Castrellón A.K., Romeo O., García-Carnero L.C., Lozoya-Pérez N.E., Mora-Montes H.M. (2020). Virulence factors in *Sporothrix schenckii*, one of the causative agents of sporotrichosis. Curr. Protein Pept. Sci..

[19] Hektoen L., Perkins C.F. (1900). Refractory subcutaneous abscesses caused by *Sporothrix schenckii*. A new pathogenic fungus. J. Exp. Med..

[20] Marimon R., Gené J., Cano J., Guarro J. (2008). Sporothrix luriei: A rare fungus from clinical origin. Med. Mycol..

[21] Barros M.B.d.L., de Almeida Paes R., Schubach A.O. (2011). *Sporothrix schenckii* and sporotrichosis. Clin. Microbiol. Rev..

[22] Lopes-Bezerra L.M., Mora-Montes H.M., Zhang Y., Nino-Vega G., Rodrigues A.M., de Camargo Z.P., de Hoog S. (2018). *Sporotrichosis between* 1898 and 2017: The evolution of knowledge on a changeable disease and on emerging etiological agents. Med. Mycol..

[23] Rasamoelina T., Maubon D., Raharolahy O., Razanakoto H., Rakotozandrindrainy N., Rakotomalala F.A., Bailly S., Sendrasoa F., Ranaivo I., Andrianarison M. (2019). Sporotrichosis in the highlands of Madagascar, 2013–2017^1^. Emerg. Infect. Dis..

[24] Tovikkai D., Maitrisathit W., Srisuttiyakorn C., Vanichanan J., Thammahong A., Suankratay C. (2020). Sporotrichosis: The case series in Thailand and literature review in Southeast Asia. Med. Mycol. Case Rep..

[25] Lopes-Bezerra L.M., Schubach A., Costa R.O. (2006). *Sporothrix schenckii* and sporotrichosis. An. Acad. Bras. Ciências.

[26] Rodriguez-Caban J., Gonzalez-Velazquez W., Perez-Sanchez L., Gonzalez-Mendez R., Rodriguez-del Valle N. (2011). Calcium/calmodulin kinase1 and its relation to thermotolerance and HSP90 in *Sporothrix schenckii*: An RNAi and yeast two-hybrid study. BMC Microbiol..

[27] Valentín-Berríos S., González-Velázquez W., Pérez-Sánchez L., González-Méndez R., Rodríguez-Del Valle N. (2009). Cytosolic phospholipase A2: A member of the signalling pathway of a new G protein α subunit in *Sporothrix schenckii*. BMC Microbiol..

[28] Díaz-Jiménez D., Pérez-García L., Martínez-Álvarez J., Mora-Montes H. (2012). Role of the fungal cell wall in pathogenesis and antifungal resistance. Curr. Fungal Infect. Rep..

[29] García-Carnero L.C., Salinas-Marín R., Lozoya-Pérez N.E., Wrobel K., Wrobel K., Martínez-Duncker I., Niño-Vega G.A., Mora-Montes H.M. (2021). The Heat shock protein 60 and Pap1 participate in the *Sporothrix schenckii*-host interaction. J. Fungi.

[30] Lopes-Bezerra L.M. (2011). *Sporothrix schenckii* cell wall peptidorhamnomannans. Front. Microbiol..

[31] López-Ramírez L.A., Martínez-Álvarez J.A., Martínez-Duncker I., Lozoya-Pérez N.E., Mora-Montes H.M. (2024). Silencing of *Sporothrix schenckii GP70* reveals its contribution to fungal adhesion, virulence, and the host-fungus interaction. J. Fungi.

[32] Gómez-Gaviria M., Martínez-Álvarez J.A., Martínez-Duncker I., Baptista A.R.S., Mora-Montes H.M. (2025). Silencing of *MNT1* and *PMT2* shows the importance of *O*-linked glycosylation during the Sporothrix schenckii-host interaction. J. Fungi.

[33] López-Ramírez L.A., Martínez-Duncker I., Márquez-Márquez A., Vargas-Macías A.P., Mora-Montes H.M. (2022). Silencing of *ROT2*, the encoding gene of the endoplasmic reticulum glucosidase II, affects the cell wall and the *Sporothrix schenckii*-host interaction. J. Fungi.

[34] Lozoya-Pérez N.E., Casas-Flores S., de Almeida J.R.F., Martínez-Álvarez J.A., López-Ramírez L.A., Jannuzzi G.P., Trujillo-Esquivel E., Estrada-Mata E., Almeida S.R., Franco B. (2019). Silencing of *OCH1* unveils the role of *Sporothrix schenckii N-*linked glycans during the host-fungus interaction. Infect. Drug Resist..

[35] Castro R.A., Kubitschek-Barreira P.H., Teixeira P.A.C., Sanches G.F., Teixeira M.M., Quintella L.P., Almeida S.R., Costa R.O., Camargo Z.P., Felipe M.S.S. (2013). Differences in cell morphometry, cell wall topography and Gp70 expression correlate with the virulence of *Sporothrix brasiliensis* clinical isolates. PLoS ONE.

[36] Rodrigues A.M., Kubitschek-Barreira P.H., Fernandes G.F., de Almeida S.R., Lopes-Bezerra L.M., de Camargo Z.P. (2015). Immunoproteomic analysis reveals a convergent humoral response signature in the *Sporothrix schenckii* complex. J. Proteom..

[37] Martínez-Álvarez J.A., García-Carnero L.C., Kubitschek-Barreira P.H., Lozoya-Pérez N.E., Belmonte-Vázquez J.L., de Almeida J.R., Antonio J.G.-I., Curty N., Villagómez-Castro J.C., Peña-Cabrera E. (2019). Analysis of some immunogenic properties of the recombinant *Sporothrix schenckii* Gp70 expressed in *Escherichia coli*. Future Microbiol..

[38] Lozoya-Pérez N.E., Clavijo-Giraldo D.M., Martínez-Duncker I., García-Carnero L.C., López-Ramírez L.A., Niño-Vega G.A., Mora-Montes H.M. (2020). Influences of the culturing media in the virulence and cell wall of *Sporothrix schenckii*, *Sporothrix brasiliensis*, and *Sporothrix globosa*. J. Fungi.

[39] Villalobos-Duno H.L., Barreto L.A., Alvarez-Aular Á., Mora-Montes H.M., Lozoya-Pérez N.E., Franco B., Lopes-Bezerra L.M., Niño-Vega G.A. (2021). Comparison of cell wall polysaccharide composition and structure between strains of *Sporothrix schenckii* and *Sporothrix brasiliensis*. Front. Microbiol..

[40] Lopes-Bezerra L.M., Walker L.A., Niño-Vega G., Mora-Montes H.M., Neves G.W.P., Villalobos-Duno H., Barreto L., Garcia K., Franco B., Martínez-Álvarez J.A. (2018). Cell walls of the dimorphic fungal pathogens *Sporothrix schenckii* and *Sporothrix brasiliensis* exhibit bilaminate structures and sloughing of extensive and intact layers. PLoS Negl. Trop. Dis..

[41] Martínez-Álvarez J.A., Pérez-García L.A., Mellado-Mojica E., López M.G., Martínez-Duncker I., Lópes-Bezerra L.M., Mora-Montes H.M. (2017). *Sporothrix schenckii sensu stricto* and *Sporothrix brasiliensis* are differentially recognized by human peripheral blood mononuclear cells. Front. Microbiol..

[42] Teixeira M.M., de Almeida L.G., Kubitschek-Barreira P., Alves F.L., Kioshima E.S., Abadio A.K., Fernandes L., Derengowski L.S., Ferreira K.S., Souza R.C. (2014). Comparative genomics of the major fungal agents of human and animal sporotrichosis: *Sporothrix schenckii* and *Sporothrix brasiliensis*. BMC Genom..

[43] Ferreira B.H., Ramírez-Prado J.H., Neves G.W.P., Torrado E., Sampaio P., Felipe M.S.S., Vasconcelos A.T., Goldman G.H., Carvalho A., Cunha C. (2019). Ploidy determination in the pathogenic fungus *Sporothrix* spp. *Front*. Microbiol..

[44] Rementeria A., López-Molina N., Ludwig A., Vivanco A.B., Bikandi J., Pontón J., Garaizar J. (2005). Genes and molecules involved in *Aspergillus fumigatus* virulence. Rev. Iberoam. Micol..

[45] Altschul S.F., Gish W., Miller W., Myers E.W., Lipman D.J. (1990). Basic local alignment search tool. J. Mol. Biol..

[46] Teixeira P.A.C., de Castro R.A., Nascimento R.C., Tronchin G., Pérez Torres A., Lazéra M., de Almeida S.R., Bouchara J.-P., Loureiro y Penha C.V., Lopes-Bezerra L.M. (2009). Cell surface expression of adhesins for fibronectin correlates with virulence in *Sporothrix schenckii*. Microbiology.

[47] Almeida-Paes R., de Oliveira L.C., Oliveira M.M.E., Gutierrez-Galhardo M.C., Nosanchuk J.D., Zancopé-Oliveira R.M. (2015). Phenotypic characteristics associated with virulence of clinical isolates from the *Sporothrix* complex. BioMed Res. Int..

[48] Lima O.C., Figueiredo C.C., Pereira B.A., Coelho M.G., Morandi V., Lopes-Bezerra L.M. (1999). Adhesion of the human pathogen *Sporothrix schenckii* to several extracellular matrix proteins. Braz. J. Med. Biol. Res..

[49] Lima O.C., Bouchara J.P., Renier G., Marot-Leblond A., Chabasse D., Lopes-Bezerra L.M. (2004). Immunofluorescence and flow cytometry analysis of fibronectin and laminin binding to *Sporothrix schenckii* yeast cells and conidia. Microb. Pathog..

[50] Ruiz-Baca E., Toriello C., Pérez-Torres A., Sabanero-López M., Villagómez-Castro J.C., López-Romero E. (2009). Isolation and some properties of a glycoprotein of 70 kDa (Gp70) from the cell wall of *Sporothrix schenckii* involved in fungal adherence to dermal extracellular matrix. Med. Mycol..

[51] Almeida S.R. (2012). Therapeutic monoclonal antibody for sporotrichosis. Front. Microbiol..

[52] Da Rosa D., Gezuele E., Calegari L., Goñi F. (2009). Excretion-secretion products and proteases from live *Sporothrix schenckii* yeast phase: Immunological detection and cleavage of human IgG. Rev. Inst. Med. Trop. Sao Paulo.

[53] Valle-Aviles L., Valentin-Berrios S., Gonzalez-Mendez R.R., Rodriguez-Del Valle N. (2007). Functional, genetic and bioinformatic characterization of a calcium/calmodulin kinase gene in *Sporothrix schenckii*. BMC Microbiol..

[54] Arvizu-Rubio V.J., García-Carnero L.C., Mora-Montes H.M. (2022). Moonlighting proteins in medically relevant fungi. PeerJ.

[55] Calderone R. (1998). The *INT1* of *Candida albicans*. Trends Microbiol..

[56] Martinez-Lopez R., Park H., Myers C.L., Gil C., Filler S.G. (2006). *Candida albicans* Ecm33p is important for normal cell wall architecture and interactions with host cells. Eukaryot. Cell.

[57] Sandini S., Stringaro A., Arancia S., Colone M., Mondello F., Murtas S., Girolamo A., Mastrangelo N., De Bernardis F. (2011). The *MP65* gene is required for cell wall integrity, adherence to epithelial cells and biofilm formation in *Candida albicans*. BMC Microbiol..

[58] Zhao W., Lü Y., Ouyang H., Zhou H., Yan J., Du T., Jin C. (2013). *N*-Glycosylation of Gel1 or Gel2 is vital for cell wall β-glucan synthesis in *Aspergillus fumigatus*. Glycobiology.

[59] Liu H., Lee M.J., Solis N.V., Phan Q.T., Swidergall M., Ralph B., Ibrahim A.S., Sheppard D.C., Filler S.G. (2016). *Aspergillus fumigatus CalA* binds to integrin α(5)β(1) and mediates host cell invasion. Nat. Microbiol..

[60] Millet N., Latgé J.P., Mouyna I. (2018). Members of glycosyl-hydrolase family 17 of *A. fumigatus* differentially affect morphogenesis. J. Fungi.

[61] Steen B.R., Zuyderduyn S., Toffaletti D.L., Marra M., Jones S.J., Perfect J.R., Kronstad J. (2003). *Cryptococcus neoformans* gene expression during experimental cryptococcal meningitis. Eukaryot. Cell.

[62] Brilhante R.S.N., de Aguiar F.R.M., da Silva M.L.Q., de Oliveira J.S., de Camargo Z.P., Rodrigues A.M., Pereira V.S., Serpa R., Castelo-Branco D., Correia E.E.M. (2018). Antifungal susceptibility of *Sporothrix schenckii* complex biofilms. Med. Mycol..

[63] Sánchez-Herrera R., Flores-Villavicencio L.L., Pichardo-Molina J.L., Castruita-Domínguez J.P., Aparicio-Fernández X., Sabanero López M., Villagómez-Castro J.C. (2021). Analysis of biofilm formation by *Sporothrix schenckii*. Med. Mycol..

[64] Dos Santos G.M.P., Borba-Santos L.P., Vila T., Ferreira Gremião I.D., Pereira S.A., De Souza W., Rozental S. (2022). *Sporothrix* spp. biofilms impact in the zoonotic transmission route: Feline claws associated biofilms, itraconazole tolerance, and potential repurposing for miltefosine. Pathogens.

[65] Zheng F., Gao W., Wang Y., Chen Q., Zhang Q., Jiang X., Hou B., Zhang Z. (2021). Map of dimorphic switching-related signaling pathways in *Sporothrix schenckii* based on its transcriptome. Mol. Med. Rep..

[66] He D., Zhang X., Gao S., You H., Zhao Y., Wang L. (2021). Transcriptome analysis of dimorphic fungus *Sporothrix schenckii* exposed to temperature stress. Int. Microbiol..

[67] Zhang Z., Hou B., Wu Y.Z., Wang Y., Liu X., Han S. (2018). Two-component histidine kinase *DRK1* is required for pathogenesis in *Sporothrix schenckii*. Mol. Med. Rep..

[68] Zheng X., Wang Y., Wang Y. (2004). Hgc1, a novel hypha-specific G1 cyclin-related protein regulates *Candida albicans* hyphal morphogenesis. Embo J..

[69] Kebaara B.W., Langford M.L., Navarathna D.H., Dumitru R., Nickerson K.W., Atkin A.L. (2008). *Candida albicans* Tup1 is involved in farnesol-mediated inhibition of filamentous-growth induction. Eukaryot. Cell.

[70] Kornitzer D. (2019). Regulation of Candida albicans Hyphal Morphogenesis by Endogenous Signals. J. Fungi.

[71] Magditch D.A., Liu T.B., Xue C., Idnurm A. (2012). DNA mutations mediate microevolution between host-adapted forms of the pathogenic fungus *Cryptococcus neoformans*. PLoS Pathog..

[72] Chadwick B.J., Pham T., Xie X., Ristow L.C., Krysan D.J., Lin X. (2022). The RAM signaling pathway links morphology, thermotolerance, and CO(2) tolerance in the global fungal pathogen *Cryptococcus neoformans*. Elife.

[73] Burnie J.P., Carter T.L., Hodgetts S.J., Matthews R.C. (2006). Fungal heat-shock proteins in human disease. FEMS Microbiol. Rev..

[74] Gong Y., Li T., Yu C., Sun S. (2017). *Candida albicans* heat shock proteins and Hsps-associated signaling pathways as potential antifungal targets. Front. Cell. Infect. Microbiol..

[75] Fabri J., Rocha M.C., Fernandes C.M., Campanella J.E.M., Cunha A.F.D., Del Poeta M., Malavazi I. (2023). The heat shock transcription factor HsfA plays a role in membrane lipids biosynthesis connecting thermotolerance and unsaturated fatty acid metabolism in *Aspergillus fumigatus*. Microbiol. Spectr..

[76] Fabri J.H.T.M., Rocha M.C., Fernandes C.M., Persinoti G.F., Ries L.N.A., Cunha A.F.d., Goldman G.H., Del Poeta M., Malavazi I. (2021). The heat shock transcription factor HsfA is essential for thermotolerance and regulates cell wall integrity in *Aspergillus fumigatus*. Front. Microbiol..

[77] Havel V.E., Wool N.K., Ayad D., Downey K.M., Wilson C.F., Larsen P., Djordjevic J.T., Panepinto J.C. (2011). Ccr4 promotes resolution of the endoplasmic reticulum stress response during host temperature adaptation in *Cryptococcus neoformans*. Eukaryot. Cell.

[78] Polke M., Hube B., Jacobsen I.D. (2015). *Candida* survival strategies. Adv. Appl. Microbiol..

[79] Sabanero López M., Flores Villavicencio L.L., Soto Arredondo K., Barbosa Sabanero G., Villagómez-Castro J.C., Cruz Jiménez G., Sandoval Bernal G., Torres Guerrero H. (2018). Proteases of *Sporothrix schenckii*: Cytopathological effects on a host-cell model. Rev. Iberoam. Micol..

[80] Carissimi M., Stopiglia C.D.O., Souza T., Corbellini V.A., Scroferneker M. (2007). Comparison of lipolytic activity of *Sporothrix schenckii* strains utilizing olive oil-rhodamine B and tween 80. Tecno Lógica.

[81] Tsuboi R., Sanada T., Takamori K., Ogawa H. (1987). Isolation and properties of extracellular proteinases from *Sporothrix schenckii*. J. Bacteriol..

[82] Gácser A., Trofa D., Schäfer W., Nosanchuk J.D. (2007). Targeted gene deletion in *Candida parapsilosis* demonstrates the role of secreted lipase in virulence. J. Clin. Investig..

[83] Lan D.M., Yang N., Wang W.K., Shen Y.F., Yang B., Wang Y.H. (2011). A novel cold-active lipase from *Candida albicans*: Cloning, expression and characterization of the recombinant enzyme. Int. J. Mol. Sci..

[84] Schaller M., Borelli C., Korting H.C., Hube B. (2005). Hydrolytic enzymes as virulence factors of *Candida albicans*. Mycoses.

[85] Ikeda M.A.K., de Almeida J.R.F., Jannuzzi G.P., Cronemberger-Andrade A., Torrecilhas A.C.T., Moretti N.S., da Cunha J.P.C., de Almeida S.R., Ferreira K.S. (2018). Extracellular vesicles from *Sporothrix brasiliensis* are an important virulence factor that induce an increase in fungal burden in experimental sporotrichosis. Front. Microbiol..

[86] Rossato L., Moreno L.F., Jamalian A., Stielow B., de Almeida S.R., de Hoog S., Freeke J. (2018). Proteins potentially involved in immune evasion strategies in *Sporothrix brasiliensis* elucidated by ultra-high-resolution mass spectrometry. Msphere.

[87] Jacobson E.S. (2000). Pathogenic roles for fungal melanins. Clin. Microbiol. Rev..

[88] Qin Y., Xia Y. (2024). Melanin in fungi: Advances in structure, biosynthesis, regulation, and metabolic engineering. Microbial Cell Factories.

[89] Almeida-Paes R., Figueiredo-Carvalho M.H.G., Brito-Santos F., Almeida-Silva F., Oliveira M.M.E., Zancopé-Oliveira R.M. (2016). Melanins protect *Sporothrix brasiliensis* and *Sporothrix schenckii* from the antifungal effects of terbinafine. PLoS ONE.

[90] Morris-Jones R., Youngchim S., Gomez B.L., Aisen P., Hay R.J., Nosanchuk J.D., Casadevall A., Hamilton A.J. (2003). Synthesis of melanin-like pigments by *Sporothrix schenckii* in vitro and during mammalian infection. Infect. Immun..

[91] Fernandes G.F., dos Santos P.O., Rodrigues A.M., Sasaki A.A., Burger E., de Camargo Z.P. (2013). Characterization of virulence profile, protein secretion and immunogenicity of different *Sporothrix schenckii sensu stricto* isolates compared with *S. globosa* and *S. brasiliensis species*. Virulence.

[92] Galván-Hernández A.K., Gómez-Gaviria M., Martínez-Duncker I., Martínez-Álvarez J.A., Mora-Montes H.M. (2023). Differential recognition of clinically relevant *Sporothrix* species by human granulocytes. J. Fungi.

[93] Lenardon M.D., Munro C.A., Gow N.A. (2010). Chitin synthesis and fungal pathogenesis. Curr. Opin. Microbiol..

[94] Martinez-Alvarez J.A., Perez-Garcia L.A., Flores-Carreon A., Mora-Montes H.M. (2014). The immune response against *Candida* spp. and *Sporothrix schenckii*. Rev. Iberoam. Micol..

[95] Kirkland T.N., Fierer J. (2020). Innate immune receptors and defense against primary pathogenic fungi. Vaccines.

[96] Gómez-Gaviria M., Martínez-Duncker I., García-Carnero L.C., Mora-Montes H.M. (2023). Differential recognition of *Sporothrix schenckii*, *Sporothrix brasiliensis*, and *Sporothrix globosa* by human monocyte-derived macrophages and dendritic cells. Infect. Drug Resist..

[97] Kischkel B., Lopes-Bezerra L., Taborda C.P., Joosten L.A.B., Dos Santos J.C., Netea M.G. (2022). Differential recognition and cytokine induction by the peptidorhamnomannan from *Sporothrix brasiliensis* and *S. schenckii*. Cell Immunol..

[98] Tamez-Castrellón A.K., van der Beek S.L., López-Ramírez L.A., Martínez-Duncker I., Lozoya-Pérez N.E., van Sorge N.M., Mora-Montes H.M. (2021). Disruption of protein rhamnosylation affects the *Sporothrix schenckii*-host interaction. Cell Surf..

[99] García-Carnero L.C., Martínez-Duncker I., Gómez-Gaviria M., Mora-Montes H.M. (2023). Differential recognition of clinically relevant *Sporothrix* species by human mononuclear cells. J. Fungi.

[100] García Carnero L.C., Lozoya Pérez N.E., González Hernández S.E., Martínez Álvarez J.A. (2018). Immunity and treatment of sporotrichosis. J. Fungi.

[101] Sassá M.F., Saturi A.E., Souza L.F., Ribeiro L.C., Sgarbi D.B., Carlos I.Z. (2009). Response of macrophage Toll-like receptor 4 to a *Sporothrix schenckii* lipid extract during experimental sporotrichosis. Immunology.

[102] Alegranci P., de Abreu Ribeiro L.C., Ferreira L.S., Negrini Tde C., Maia D.C., Tansini A., Gonçalves A.C., Placeres M.C., Carlos I.Z. (2013). The predominance of alternatively activated macrophages following challenge with cell wall peptide-polysaccharide after prior infection with *Sporothrix schenckii*. Mycopathologia.

[103] Negrini T.d.C., Ferreira L.S., Alegranci P., Arthur R.A., Sundfeld P.P., Maia D.C.G., Spolidorio L.C., Carlos I.Z. (2013). Role of TLR-2 and fungal surface antigens on innate immune response against *Sporothrix schenckii*. Immunol. Investig..

[104] Sassá M.F., Ferreira L.S., Ribeiro L.C., Carlos I.Z. (2012). Immune response against *Sporothrix schenckii* in TLR-4-deficient mice. Mycopathologia.

[105] Reid D.M., Gow N.A., Brown G.D. (2009). Pattern recognition: Recent insights from Dectin-1. Curr. Opin. Immunol..

[106] Franco Dde L., Nascimento R.C., Ferreira K.S., Almeida S.R. (2012). Antibodies against *Sporothrix schenckii* enhance TNF-α production and killing by macrophages. Scand. J. Immunol..

[107] Huang L., Zhang J., Du W., Liang Z., Li M., Wu R., Chen S., Hu X., Huang H. (2021). Chitin-rich heteroglycan from *Sporothrix schenckii sensu stricto* potentiates fungal clearance in a mouse model of sporotrichosis and promotes macrophages phagocytosis. BMC Microbiol..

[108] Maia D.C., Gonçalves A.C., Ferreira L.S., Manente F.A., Portuondo D.L., Vellosa J.C., Polesi M.C., Batista-Duharte A., Carlos I.Z. (2016). Response of cytokines and hydrogen peroxide to *Sporothrix schenckii* exoantigen in systemic experimental infection. Mycopathologia.

[109] Fernandes K.S., Neto E.H., Brito M.M., Silva J.S., Cunha F.Q., Barja-Fidalgo C. (2008). Detrimental role of endogenous nitric oxide in host defence against Sporothrix schenckii. Immunology.

[110] Romero-Martinez R., Wheeler M., Guerrero-Plata A., Rico G., Torres-Guerrero H. (2000). Biosynthesis and functions of melanin in *Sporothrix schenckii*. Infect. Immun..

[111] Guzman-Beltran S., Perez-Torres A., Coronel-Cruz C., Torres-Guerrero H. (2012). Phagocytic receptors on macrophages distinguish between different *Sporothrix schenckii* morphotypes. Microbes Infect..

[112] de Miranda L.H.M., Santiago M.A., Frankenfeld J., Reis E.G.D., Menezes R.C., Pereira S.A., Gremião I.D.F., Hofmann-Lehmann R., Conceição-Silva F. (2024). Neutrophil oxidative burst profile is related to a satisfactory response to itraconazole and clinical cure in feline sporotrichosis. J. Fungi.

[113] Verdan F.F., Faleiros J.C., Ferreira L.S., Monnazzi L.G., Maia D.C., Tansine A., Placeres M.C., Carlos I.Z., Santos-Junior R.R. (2012). Dendritic cell are able to differentially recognize *Sporothrix schenckii* antigens and promote Th1/Th17 response in vitro. Immunobiology.

[114] Uenotsuchi T., Takeuchi S., Matsuda T., Urabe K., Koga T., Uchi H., Nakahara T., Fukagawa S., Kawasaki M., Kajiwara H. (2006). Differential induction of Th1-prone immunity by human dendritic cells activated with *Sporothrix schenckii* of cutaneous and visceral origins to determine their different virulence. Int. Immunol..

[115] Sgarbi D.B., da Silva A.J., Carlos I.Z., Silva C.L., Angluster J., Alviano C.S. (1997). Isolation of ergosterol peroxide and its reversion to ergosterol in the pathogenic fungus *Sporothrix schenckii*. Mycopathologia.

[116] Remer K.A., Brcic M., Jungi T.W. (2003). Toll-like receptor-4 is involved in eliciting an LPS-induced oxidative burst in neutrophils. Immunol. Lett..

[117] de Almeida J.R., Kaihami G.H., Jannuzzi G.P., de Almeida S.R. (2015). Therapeutic vaccine using a monoclonal antibody against a 70-kDa glycoprotein in mice infected with highly virulent *Sporothrix schenckii* and *Sporothrix brasiliensis*. Med. Mycol..

[118] Portuondo D.L., Batista-Duharte A., Ferreira L.S., Martínez D.T., Polesi M.C., Duarte R.A., de Paula E.S.A.C., Marcos C.M., Almeida A.M., Carlos I.Z. (2016). A cell wall protein-based vaccine candidate induce protective immune response against *Sporothrix schenckii* infection. Immunobiology.

[119] Alba-Fierro C.A., Pérez-Torres A., López-Romero E., Cuéllar-Cruz M., Ruiz-Baca E. (2014). Cell wall proteins of *Sporothrix schenckii* as immunoprotective agents. Rev. Iberoam. Micol..

[120] García-Carnero L.C., Pérez-García L.A., Martínez-Álvarez J.A., Reyes-Martínez J.E., Mora-Montes H.M. (2018). Current trends to control fungal pathogens: Exploiting our knowledge in the host-pathogen interaction. Infect. Drug Resist..

[121] Bonifaz A., Vázquez-González D. (2013). Diagnosis and treatment of lymphocutaneous sporotrichosis: What are the options?. Curr. Fungal Infect. Rep..

[122] Zhang Y., Hagen F., Stielow B., Rodrigues A.M., Samerpitak K., Zhou X., Feng P., Yang L., Chen M., Deng S. (2015). Phylogeography and evolutionary patterns in *Sporothrix* spanning more than 14 000 human and animal case reports. Persoonia.

[123] Rodrigues A.M., de Hoog G.S., de Camargo Z.P. (2016). *Sporothrix* species causing outbreaks in animals and humans driven by animal-animal transmission. PLoS Pathog..

[124] Gremião I.D.F., Miranda L.H.M., Reis E.G., Rodrigues A.M., Pereira S.A. (2017). Zoonotic epidemic of sporotrichosis: Cat to human transmission. PLoS Pathog..

[125] Rabello V.B.S., Almeida M.A., Bernardes-Engemann A.R., Almeida-Paes R., de Macedo P.M., Zancopé-Oliveira R.M. (2022). The historical burden of sporotrichosis in Brazil: A systematic review of cases reported from 1907 to 2020. Braz. J. Microbiol..

[126] Govender N.P., Maphanga T.G., Zulu T.G., Patel J., Walaza S., Jacobs C., Ebonwu J.I., Ntuli S., Naicker S.D., Thomas J. (2015). An outbreak of lymphocutaneous sporotrichosis among mine-workers in South Africa. PLoS Negl. Trop. Dis..

[127] Hardman S., Stephenson I., Jenkins D.R., Wiselka M.J., Johnson E.M. (2005). Disseminated *Sporothix schenckii* in a patient with AIDS. J. Infect..

[128] Silva-Vergara M.L., Maneira F.R., De Oliveira R.M., Santos C.T., Etchebehere R.M., Adad S.J. (2005). Multifocal sporotrichosis with meningeal involvement in a patient with AIDS. Med. Mycol..

[129] Carvalho M.T., de Castro A.P., Baby C., Werner B., Filus Neto J., Queiroz-Telles F. (2002). Disseminated cutaneous sporotrichosis in a patient with AIDS: Report of a case. Rev. Soc. Bras. Med. Trop..

[130] Almeida H.L., Lettnin C.B., Barbosa J.L., Dias M.C. (2009). Spontaneous resolution of zoonotic sporotrichosis during pregnancy. Rev. Inst. Med. Trop. Sao Paulo.

[131] Lederer H.T., Sullivan E., Crum-Cianflone N.F. (2016). Sporotrichosis as an unusual case of osteomyelitis: A case report and review of the literature. Med. Mycol. Case Rep..

[132] Pappas P.G., Tellez I., Deep A.E., Nolasco D., Holgado W., Bustamante B. (2000). Sporotrichosis in Peru: Description of an area of hyperendemicity. Clin. Infect. Dis..

[133] Mata-Essayag S., Delgado A., Colella M.T., Landaeta-Nezer M.E., Rosello A., Perez de Salazar C., Olaizola C., Hartung C., Magaldi S., Velasquez E. (2013). Epidemiology of sporotrichosis in Venezuela. Int. J. Dermatol.

[134] Tai F., Jakubovic H., Alabdulrazzaq S., Alavi A. (2020). A case of sporotrichosis infection mimicking pyoderma gangrenosum and the role of tissue culture in diagnosis: A case report. SAGE Open Med. Case Rep..

[135] Toriello C., Brunner-Mendoza C., Ruiz-Baca E., Duarte-Escalante E., Pérez-Mejía A., Del Rocío Reyes-Montes M. (2021). Sporotrichosis in Mexico. Braz. J. Microbiol..

[136] Álvarez-Acevedo L.C., Zuleta-González M.C., Gómez-Guzmán Ó.M., Rúa-Giraldo Á.L., Hernández-Ruiz O., McEwen-Ochoa J.G., Urán-Jiménez M.E., Arango-Arteaga M., Zancopé-Oliveira R.M., Evangelista de Oliveira M.M. (2023). Phenotypic and genotypic characterization of Colombian clinical isolates of *Sporothrix* spp.. Biomedica.

[137] Zhou X., Rodrigues A.M., Feng P., Hoog G.S. (2013). Global ITS diversity in the Sporothrix schenckii complex. Fungal Divers..

[138] Dooley D.P., Bostic P.S., Beckius M.L. (1997). Spook house sporotrichosis: A point-source outbreak of sporotrichosis associated with hay bale props in a halloween haunted house. Arch. Intern. Med..

[139] Bonifaz A., Morales-Peña N., Tirado-Sánchez A., Jiménez-Mendoza D.R., Treviño-Rangel R.J., González G.M. (2020). Atypical sporotrichosis related to *Sporothrix mexicana*. Mycopathologia.

[140] Bonifaz A., Tirado-Sánchez A., Paredes-Solís V., Cepeda-Valdés R., González G.M., Treviño-Rangel R.J., Fierro-Arias L. (2018). Cutaneous disseminated sporotrichosis: Clinical experience of 24 cases. J. Eur. Acad. Dermatol. Venereol..

[141] Martínez-Duncker I., Mayorga-Rodríguez J., Gómez-Gaviria M., Martínez-Álvarez J.A., Baruch-Martínez D.A., López-Ramírez L.A., Mora-Montes H.M. (2025). Phenotypic immunological profiling and antifungal susceptibility of *Sporothrix schenckii* clinical isolates from a hyperendemic region in western Mexico. Med. Mycol..

[142] Fukushiro R. (1984). Epidemiology and ecology of sporotrichosis in Japan. Zentralbl Bakteriol. Mikrobiol. Hyg. A.

[143] Chieosilapatham P., Chuamanochan M., Chiewchavit S., Saikruatep R., Amornrungsun E., Preechasuth K. (2023). *Sporothrix schenckii sensu stricto* related to zoonotic transmission in Thailand. Med. Mycol. Case Rep..

[144] Jin W., Liu Y., Ning Q., Wu S., Su S., Zheng D., Ma S., Zou J., Yang M., Hu D. (2024). A case of chronic wounds caused by *Sporothrix schenckii* infection was rapidly detected by metagenomic next generation sequencing. Heliyon.

[145] Cheng S., Zheng S., Zhong M., Gyawali K.R., Pan W., Xu M., Huang H., Huang X. (2024). Current situation of sporotrichosis in China. Future Microbiol..

[146] Li J., Zhan P., Jiang Q., Gao Y., Jin Y., Zhang L., Luo Y., Fan X., Sun J., de Hoog S. (2019). Prevalence and antifungal susceptibility of Sporothrix species in Jiangxi, central China. Med. Mycol..

[147] Mehta K.I., Sharma N.L., Kanga A.K., Mahajan V.K., Ranjan N. (2007). Isolation of *Sporothrix schenckii* from the environmental sources of cutaneous sporotrichosis patients in Himachal Pradesh, India: Results of a pilot study. Mycoses.

[148] Reinprayoon U., Jermjutitham M., Tirakunwichcha S., Banlunara W., Tulvatana W., Chindamporn A. (2020). Conjunctival sporotrichosis from cat to human: Case report. Am. J. Ophthalmol. Case Rep..

[149] Tang M.M., Tang J.J., Gill P., Chang C.C., Baba R. (2012). Cutaneous sporotrichosis: A six-year review of 19 cases in a tertiary referral center in Malaysia. Int. J. Dermatol..

[150] Kamal Azam N.K., Selvarajah G.T., Santhanam J., Abdul Razak M.F., Ginsapu S.J., James J.E., Suetrong S. (2020). Molecular epidemiology of *Sporothrix schenkii* isolates in Malaysia. Med. Mycol..

[151] Vismer H.F., Hull P.R. (1997). Prevalence, epidemiology and geographical distribution of *Sporothrix schenckii* infections in Gauteng, South Africa. Mycopathologia.

[152] Marimon R., Gené J., Cano J., Trilles L., Dos Santos Lazéra M., Guarro J. (2006). Molecular phylogeny of *Sporothrix schenckii*. J. Clin. Microbiol..

[153] Criseo G., Romeo O. (2010). Ribosomal DNA sequencing and phylogenetic analysis of environmental *Sporothrix schenckii* strains: Comparison with clinical isolates. Mycopathologia.

[154] Romeo O., Scordino F., Criseo G. (2011). New insight into molecular phylogeny and epidemiology of *Sporothrix schenckii* species complex based on calmodulin-encoding gene analysis of Italian isolates. Mycopathologia.

[155] Morgado D.S., Castro R., Ribeiro-Alves M., Corrêa-Moreira D., Castro-Alves J., Pereira S.A., Menezes R.C., Oliveira M.M.E. (2022). Global distribution of animal sporotrichosis: A systematic review of *Sporothrix* sp. identified using molecular tools. Curr. Res. Microb. Sci..

[156] Morgado D.S., Castro R., Ribeiro-Alves M., Corrêa-Moreira D., Silva J., Menezes R.C., Oliveira M.M.E. (2024). Systematic review of literature to evaluate global distribution of species of the *Sporothrix* genus stored in culture collections. Front. Cell Infect. Microbiol..

[157] Rodrigues A.M., Gonçalves S.S., de Carvalho J.A., Borba-Santos L.P., Rozental S., Camargo Z.P. (2022). d. Current progress on epidemiology, diagnosis, and treatment of sporotrichosis and their future trends. J. Fungi.

[158] Saeed L., Weber R.J., Puryear S.B., Bahrani E., Peluso M.J., Babik J.M., Haemel A., Coates S.J. (2019). Disseminated cutaneous and osteoarticular sporotrichosis mimicking pyoderma gangrenosum. Open Forum Infect. Dis..

[159] Sendrasoa F.A., Ranaivo I.M., Sata M., Razanakoto N.H., Andrianarison M., Ratovonjanahary V., Raharolahy O., Rakotoarisaona M., Rasamoelina T., Andrianarivelo M.R. (2021). Osteoarticular sporotrichosis in an immunocompetent patient. Med. Mycol. Case Rep..

[160] Santos A., Mota A.C.O., Jesus G.R., Rocha M.D.G., Durço D., Rezende L., Silva A., Vilar F.C., Bollela V.R., Martinez R. (2024). Disseminated sporotrichosis with osteoarticular involvement in a patient with acquired immunodeficiency syndrome: A case report. Rev. Soc. Bras. Med. Trop..

[161] Schubach T.M., de Oliveira Schubach A., dos Reis R.S., Cuzzi-Maya T., Blanco T.C., Monteiro D.F., Barros B.M., Brustein R., Zancopé-Oliveira R.M., Fialho Monteiro P.C. (2002). *Sporothrix schenckii* isolated from domestic cats with and without sporotrichosis in Rio de Janeiro, Brazil. Mycopathologia.

[162] Duangkaew L., Yurayart C., Limsivilai O., Chen C., Kasorndorkbua C. (2019). Cutaneous sporotrichosis in a stray cat from Thailand. Med. Mycol. Case Rep..

[163] Han H.S., Kano R. (2021). Feline sporotrichosis in Asia. Braz. J. Microbiol..

[164] Gremião I.D., Menezes R.C., Schubach T.M., Figueiredo A.B., Cavalcanti M.C., Pereira S.A. (2015). Feline sporotrichosis: Epidemiological and clinical aspects. Med. Mycol..

[165] Miranda L., Gillett S., Ames Y., Krockenberger M., Malik R. (2024). Zoonotic feline sporotrichosis: A small case cluster in Perth, Western Australia, and a review of previous feline cases from Australia. Aust. Vet. J..

[166] Pereira S.A., Passos S.R., Silva J.N., Gremião I.D., Figueiredo F.B., Teixeira J.L., Monteiro P.C., Schubach T.M. (2010). Response to azolic antifungal agents for treating feline sporotrichosis. Vet. Rec..

[167] de Miranda L.H.M., Silva J.N., Gremião I.D.F., Menezes R.C., Almeida-Paes R., Dos Reis É.G., de Oliveira R.V.C., de Araujo D., Ferreiro L., Pereira S.A. (2018). Monitoring fungal burden and viability of *Sporothrix* spp. in skin lesions of cats for predicting antifungal treatment response. J. Fungi.

[168] Mothé G., Reis N., Melivilu C., Junior A., Santos C., Dieckmann A., Dantas Machado R.L., Rocha E., Baptista A. (2021). Ocular lesions in a domestic feline:: A closer look at the fungal pathogen *Sporothrix brasiliensis*. Braz. J. Vet. Res. Anim. Sci..

[169] Schubach T.M., Schubach A., Okamoto T., Barros M.B., Figueiredo F.B., Cuzzi T., Fialho-Monteiro P.C., Reis R.S., Perez M.A., Wanke B. (2004). Evaluation of an epidemic of sporotrichosis in cats: 347 cases (1998–2001). J. Am. Vet. Med. Assoc..

[170] Gonzalez Cabo J.F., de las Heras Guillamon M., Latre Cequiel M.V., Garcia de Jalon Ciercoles J.A. (1989). Feline sporotrichosis: A case report. Mycopathologia.

[171] Kano R., Okubo M., Siew H.H., Kamata H., Hasegawa A. (2015). Molecular typing of *Sporothrix schenckii* isolates from cats in Malaysia. Mycoses.

[172] Siew H.H. (2017). The current status of feline sporotrichosis in Malaysia. Med. Mycol. J..

[173] Hennessee I., Barber E., Petro E., Lindemann S., Buss B., Santos A., Gade L., Lockhart S.R., Sexton D.J., Chiller T. (2024). Sporotrichosis cluster in domestic cats and veterinary technician, Kansas, USA, 2022. Emerg. Infect. Dis..

[174] Nakamura Y., Sato H., Watanabe S., Takahashi H., Koide K., Hasegawa A. (1996). *Sporothrix schenckii* isolated from a cat in Japan. Mycoses.

[175] Silva B.W.L., Maués M.A.C., Barrozo P.H.M., Brito J.d.S., de Souza C.C.N., Rosário M.K.S.D., Santos C.D.S.B., Neta A.A.M.Q., Martins F.M.S., da Silva L.B.G. (2022). First report of fungal *Sporothrix schenckii* complex isolation from feline with possible zoonotic transmission in the city of Belém, Pará, Brazil: Case report. Res. Soc. Dev..

[176] Schubach T.M., Schubach A., Okamoto T., Barros M.B., Figueiredo F.B., Cuzzi T., Pereira S.A., Dos Santos I.B., Almeida Paes R., Paes Leme L.R. (2006). Canine sporotrichosis in Rio de Janeiro, Brazil: Clinical presentation, laboratory diagnosis and therapeutic response in 44 cases (1998–2003). Med. Mycol..

[177] Cafarchia C., Sasanelli M., Lia R.P., de Caprariis D., Guillot J., Otranto D. (2007). Lymphocutaneous and nasal sporotrichosis in a dog from southern Italy: Case report. Mycopathologia.

[178] Viana P.G., Figueiredo A.B.F., Gremião I.D.F., de Miranda L.H.M., da Silva Antonio I.M., Boechat J.S., de Sá Machado A.C., de Oliveira M.M.E., Pereira S.A. (2018). Successful treatment of canine sporotrichosis with terbinafine: Case reports and literature review. Mycopathologia.

[179] Prazeres Júnior F.R., Moreira A.C., Medeiros N.O., Carmo M.C.C., Lima M.P.S. (2024). Sporotrichosis in guinea pig (*Cavia porcellu*)—Case report. Arq. Bras. Med. Veterinária E Zootec..

[180] Mahmoudi S., Zaini F. (2015). Sporotrichosis in Iran: A mini review of reported cases in patients suspected to cutaneous leishmaniasis. Curr. Med. Mycol..

[181] De Carolis E., Posteraro B., Sanguinetti M. (2022). Old and new insights into *Sporothrix schenckii* complex biology and identification. Pathogens.

[182] Oliveira M.M., Almeida-Paes R., Gutierrez-Galhardo M.C., Zancope-Oliveira R.M. (2014). Molecular identification of the *Sporothrix schenckii* complex. Rev. Iberoam. Micol..

[183] Hu S., Chung W.H., Hung S.I., Ho H.C., Wang Z.W., Chen C.H., Lu S.C., Kuo T.T., Hong H.S. (2003). Detection of *Sporothrix schenckii* in clinical samples by a nested PCR assay. J. Clin. Microbiol..

[184] Arenas R., Miller D., Campos-Macias P. (2007). Epidemiological data and molecular characterization (mtDNA) of *Sporothrix schenckii* in 13 cases from Mexico. Int. J. Dermatol..

[185] de Oliveira M.M., Sampaio P., Almeida-Paes R., Pais C., Gutierrez-Galhardo M.C., Zancope-Oliveira R.M. (2012). Rapid identification of *Sporothrix* species by T3B fingerprinting. J. Clin. Microbiol..

[186] Zhang M., Li F., Gong J., Yang X., Zhang J., Zhao F. (2020). Development and evaluation of a real-time polymerase chain reaction for fast diagnosis of sporotrichosis caused by *Sporothrix globosa*. Med. Mycol..

[187] Oliveira M.M., Santos C., Sampaio P., Romeo O., Almeida-Paes R., Pais C., Lima N., Zancopé-Oliveira R.M. (2015). Development and optimization of a new MALDI-TOF protocol for identification of the *Sporothrix* species complex. Res. Microbiol..

[188] Mahajan V.K. (2014). Sporotrichosis: An overview and therapeutic options. Dermatol. Res. Pract..

[189] Kauffman C.A., Bustamante B., Chapman S.W., Pappas P.G. (2007). Clinical practice guidelines for the management of sporotrichosis: 2007 update by the Infectious Diseases Society of America. Clin. Infect. Dis..

[190] Kohler L.M., Soares B.M., de Assis Santos D., Da Silva Barros M.E., Hamdan J.S. (2006). *In vitro* susceptibility of isolates of *Sporothrix schenckii* to amphotericin B, itraconazole, and terbinafine: Comparison of yeast and mycelial forms. Can. J. Microbiol..

[191] Borba-Santos L.P., Rodrigues A.M., Gagini T.B., Fernandes G.F., Castro R., de Camargo Z.P., Nucci M., Lopes-Bezerra L.M., Ishida K., Rozental S. (2015). Susceptibility of *Sporothrix brasiliensis* isolates to amphotericin B, azoles, and terbinafine. Med. Mycol..

[192] Chapman S.W., Pappas P., Kauffmann C., Smith E.B., Dietze R., Tiraboschi-Foss N., Restrepo A., Bustamante A.B., Opper C., Emady-Azar S. (2004). Comparative evaluation of the efficacy and safety of two doses of terbinafine (500 and 1000 mg day(^−1^)) in the treatment of cutaneous or lymphocutaneous sporotrichosis. Mycoses.

[193] Francesconi G., Francesconi do Valle A.C., Passos S.L., de Lima Barros M.B., de Almeida Paes R., Curi A.L., Liporage J., Porto C.F., Galhardo M.C. (2011). Comparative study of 250 mg/day terbinafine and 100 mg/day itraconazole for the treatment of cutaneous sporotrichosis. Mycopathologia.

[194] Yamada K., Zaitz C., Framil V.M., Muramatu L.H. (2011). Cutaneous sporotrichosis treatment with potassium iodide: A 24 year experience in São Paulo State, Brazil. Rev. Inst. Med. Trop. Sao Paulo.

[195] Macedo P.M., Lopes-Bezerra L.M., Bernardes-Engemann A.R., Orofino-Costa R. (2015). New posology of potassium iodide for the treatment of cutaneous sporotrichosis: Study of efficacy and safety in 102 patients. J. Eur. Acad. Dermatol. Venereol..

[196] Bustamante B., Campos P.E. (2001). Endemic sporotrichosis. Curr. Opin. Infect. Dis..

[197] Borba-Santos L.P., Vila T., Rozental S. (2020). Identification of two potential inhibitors of *Sporothrix brasiliensis* and *Sporothrix schenckii* in the Pathogen Box collection. PLoS ONE.

[198] Aroonvuthiphong V., Bangphoomi N. (2025). Therapeutic alternatives for sporotrichosis induced by wild-type and non-wild-type *Sporothrix schenckii* through in vitro and in vivo assessment of enilconazole, isavuconazole, posaconazole, and terbinafine. Sci. Rep..

[199] Masoko P., Picard J., Howard R.L., Mampuru L.J., Eloff J.N. (2010). *In vivo* antifungal effect of *Combretum* and *Terminalia* species extracts on cutaneous wound healing in immunosuppressed rats. Pharm. Biol..

[200] Freitas D.F., de Siqueira Hoagland B., do Valle A.C., Fraga B.B., de Barros M.B., de Oliveira Schubach A., de Almeida-Paes R., Cuzzi T., Rosalino C.M., Zancopé-Oliveira R.M. (2012). Sporotrichosis in HIV-infected patients: Report of 21 cases of endemic sporotrichosis in Rio de Janeiro, Brazil. Med. Mycol..

[201] Pinto-Almazán R., Sandoval-Navarro K.A., Damián-Magaña E.J., Arenas R., Fuentes-Venado C.E., Zárate-Segura P.B., Martínez-Herrera E., Rodríguez-Cerdeira C. (2023). Relationship of sporotrichosis and infected patients with HIV-AIDS: An actual systematic review. J. Fungi.

[202] de Almeida J.R., Santiago K.L., Kaihami G.H., Maranhão A.Q., de Macedo Brígido M., de Almeida S.R. (2017). The efficacy of humanized antibody against the *Sporothrix* antigen, gp70, in promoting phagocytosis and reducing disease burden. Front. Microbiol..

[203] de Almeida S.R. (2019). Advances in vaccine development against sporotrichosis. Curr. Trop. Med. Rep..

[204] Rodrigues A.M., Della Terra P.P., Gremião I.D., Pereira S.A., Orofino-Costa R., de Camargo Z.P. (2020). The threat of emerging and re-emerging pathogenic *Sporothrix* species. Mycopathologia.

